# Supplementary figures and images for: A multiscale electro-metabolic model of a rat neocortical circuit reveals the impact of ageing on central cortical layers
Source: PLoS Comput Biol. 2025 May 20;21(5):e1013070. doi: 10.1371/journal.pcbi.1013070 (PMC12112163; doi:10.1371/journal.pcbi.1013070)

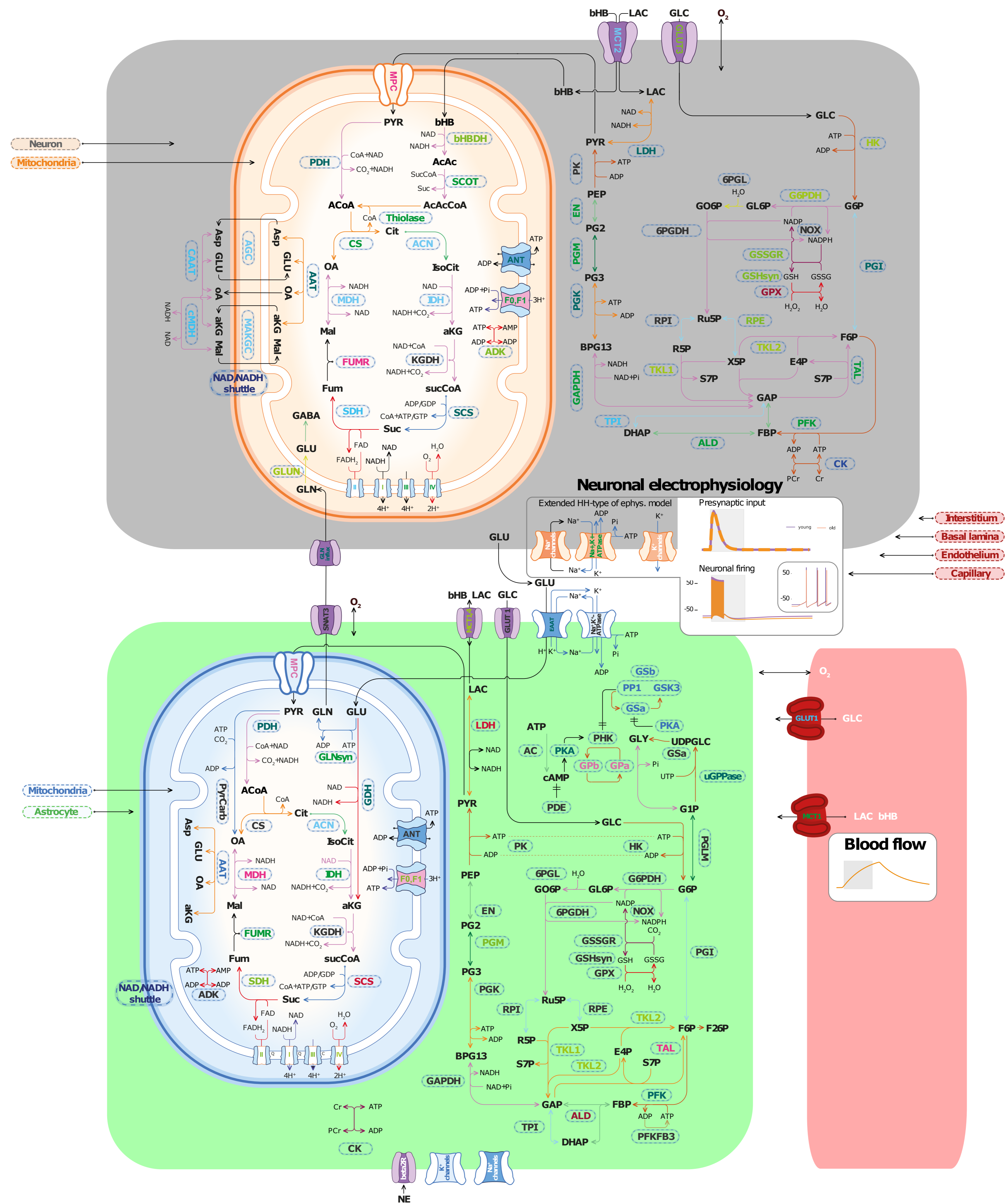

Supplement: S1 Fig — (PDF) [file pcbi.1013070.s001.pdf]
