# Supplementary material for: A multiscale electro-metabolic model of a rat neocortical circuit reveals the impact of ageing on central cortical layers
Source: PLoS Comput Biol. 2025 May 20;21(5):e1013070. doi: 10.1371/journal.pcbi.1013070 (PMC12112163; doi:10.1371/journal.pcbi.1013070)
Supplement: S1 Text — Table A: m-types used in the circuit and their descriptions. Table B: model mechanisms and their respective parameters used for constructing e-models. Table C: mechanisms and their neuron model locations for different e-types. Table D: parameters of the electrical model used in single neuron models and optimization. (PDF) [file pcbi.1013070.s002.pdf]

S1 Text: A multiscale electro-metabolic model of a rat neocortical circuit reveals the impact of ageing on central cortical layers

Sofia Farina[1], Alessandro Cattabiani[1], Darshan Mandge[1], Polina Shichkova[1,2], James B. Isbister[1], Jean Jacquemier[1], James G. King[1], Henry Markram[1,3], Daniel Keller[1]

Table A: m-types used in the circuit and their descriptions.

m-type	Description	m-type	Description
L1_DAC	Layer 1, Descending Axon Cell	L4_TPC	Layer 4, Tufted Pyramidal Cell
L1_LAC	Layer 1, Large Axon Cell	L4_UPC	Layer 4, Untufted Pyramidal Cell
L1_HAC	Layer 1, Horizontal Axon Cell	L5_BP	Layer 5, Bipolar Cell
L1_NGC-DA	Layer 1, Neurogliaform Cell (Dense Axon Arbours)	L5_BTC	Layer 5, Bitufted Cell
L1_NGC-SA	Layer 1, Neurogliaform Cell (Sparse Axon Arbours)	L5_CHC	Layer 5, Chandelier Cell
L1_SAC	Layer 1, Small Axon Cell	L5_DBC	Layer 5, Double Bouquet Cell
L2_IPC	Layer 2, Inverted Pyramidal Cell	L5_LBC	Layer 5, Large Basket Cell
L2_TPC:A	Layer 2, Large Tufted Pyramidal Cell	L5_MC	Layer 5, Martinotti Cell
L2_TPC:B	Layer 2, Early Bifurcating Tufted Pyramidal Cell	L5_NBC	Layer 5, Nest Basket Cell
L23_BP	Layer 2/3, Bipolar Cell	L5_SBC	Layer 5, Small Basket Cell
L23_BTC	Layer 2/3, Bitufted Cell	L5_TPC:A	Layer 5, Large Tufted Pyramidal Cell
L23_CHC	Layer 2/3, Chandelier Cell	L5_TPC:B	Layer 5, Early Bifurcating Tufted Pyramidal Cell
L23_DBC	Layer 2/3, Double Bouquet Cell	L5_TPC:C	Layer 5, Small Tufted Pyramidal Cell
L23_LBC	Layer 2/3, Large Basket Cell	L5_UPC	Layer 5, Untufted Pyramidal Cell
L23_MC	Layer 2/3, Martinotti Cell	L6_BPC	Layer 6, Bitufted Pyramidal Cell
L23_NBC	Layer 2/3, Nest Basket Cell	L6_BTC	Layer 6, Bitufted Cell
L23_NGC	Layer 2/3, Neurogliaform Cell	L6_CHC	Layer 6, Chandelier Cell
L23_SBC	Layer 2/3, Small Basket Cell	L6_DBC	Layer 6, Double Bouquet Cell
L3_TPC:A	Layer 3, Large Tufted Pyramidal Cell	L6_HPC	Layer 6, Horizontal Pyramidal Cell
L3_TPC:C	Layer 3, Small Tufted Pyramidal Cell	L6_IPC	Layer 6, Inverted Pyramidal Cell
L4_BP	Layer 4, Bipolar Cell	L6_LBC	Layer 6, Large Basket Cell
L4_BTC	Layer 4, Bitufted Cell	L6_MC	Layer 6, Martinotti Cell
L4_DBC	Layer 4, Double Bouquet Cell	L6_NBC	Layer 6, Nest Basket Cell
L4_LBC	Layer 4, Large Basket Cell L6_NGC	Layer 6, Neurogliaform Cell	
L4_MC	Layer 4, Martinotti Cell	L6_SBC	Layer 6, Small Basket Cell
L4_NBC	Layer 4, Nest Basket Cell	L6_TPC:A	Layer 6, Large Tufted Pyramidal Cell
L4_SBC	Layer 4, Small Basket Cell	L6_TPC:C	Layer 6, Small Tufted Pyramidal Cell
L4_SSC	Layer 4, Spiny Stellate Cell	L6_UPC	Layer 6, Untufted Pyramidal Cell
L4_CHC	Layer 4, Chandelier Cell	L4_NGC	Layer 4, Neurogliaform Cell
L5_NGC	Layer 4, Neurogliaform Cell	L6_BP	Layer 5, Bipolar Cell

Table B: model mechanisms and their respective parameters used for constructing e-models.

Mechanism (SUFFIX)	Parameter abbreviation	Reference
**Sodium (Na) Channels**		
Transient Na (NaTg)	gNaTgbar_NaTg (maximum conductance)	[1]
Persistent Na (Nap_Et2)	gNap_Et2bar_Nap_Et2 (maximum conductance)	[2, 3]
**Potassium (K) Channels**		
Transient K (K_Tst)	gK_Tstbar_K_Tst (maximum conductance)	[4]
Persistent K (K_Pst)	gK_Pstbar_K_Pst (maximum conductance)	[4]
Kv3.1 (SKv3_1)	gSKv3_1bar_SKv3_1 (maximum conductance)	[5, 6]
D-type K (KdShu2007)	gkbar_KdShu2007 (maximum conductance)	[7]
Stochastic K (StochKv3)	gkbar_StochKv3 (maximum conductance)	[8, 9]
SKCa (SK_E2)	gSK_E2bar_SK_E2 (maximum conductance)	[10]
**Calcium (Ca) Channels**		
HVA Ca (Ca_HVA2)	gCa_HVAbar_Ca_HVA2 (maximum conductance)	[11–13]
LVA Ca (Ca_LVAst)	gCa_LVAstbar_Ca_LVAst (maximum conductance)	[14, 15]
Hyperpolarisation-activated (Ih)	gIhbar_Ih (maximum conductance)	[16, 17]
**Ion Dynamics** (internal_ions)	decay_internal_ions (Ca decay constant), gamma_internal_ions (percentage of free Ca)	
**Leak** (leak)	g_leak (maximum conductance)	[18]
**Na-K Pump** (nakpump)	totalpump_nakpump (pump density)	[19]
**Co-transporters**		
K-Cl Co-transporter (kcc2)	gKCC_kcc2 (maximum conductance)	[20]
Na-K-Cl Co-transporter (nakcc)	gNKCC_nakcc (maximum conductance)	

Table C: mechanisms and their neuron model locations for different e-types.

e-type	Myelinated Axon	AIS	Soma	Apical Dendrite	Basal Dendrite
cADpyr	Leak<br>Internal Ions	Transient Na<br>Persistent Na<br>Transient K<br>Persistent K<br>Kv3.1<br>SKCa<br>HVA Ca<br>LVA Ca<br>Na-K Co-transporter<br>Na-K Pump<br>Leak<br>Internal Ions	Transient Na<br>Transient K<br>Persistent K<br>Kv3.1<br>SKCa<br>HVA Ca<br>LVA Ca<br>Hyperpolarisation activated<br>Na-K Co-transporter<br>Na-K Pump<br>K-Cl Cotransporter 2<br>Leak<br>Internal Ions	Transient Na<br>Kv3.1<br>SKCa<br>HVA Ca<br>LVA Ca<br>Hyperpolarisation activated<br>Na-K Co-transporter<br>Na-K Pump<br>K-Cl Cotransporter 2<br>Leak<br>Internal Ions	HVA Ca<br>LVA Ca<br>Hyperpolarisation activated<br>Na-K Co-transporter<br>Na-K Pump<br>K-Cl Cotransporter 2<br>Leak<br>Internal Ions
bAC<br>bNAC<br>cACint<br>cNAC	Leak<br>Internal Ions	Persistent Na<br>Transient K<br>Persistent K<br>Kv3.1<br>SKCa<br>HVA Ca<br>LVA Ca<br>Na-K Co-transporter<br>Na-K Pump<br>Leak<br>Internal Ions	Transient K<br>Persistent K<br>Kv3.1<br>SKCa<br>HVA Ca<br>LVA Ca<br>Hyperpolarisation activated<br>Na-K Co-transporter<br>Na-K Pump<br>K-Cl Cotransporter 2<br>Leak<br>Internal Ions		HVA Ca<br>LVA Ca<br>Hyperpolarisation activated<br>Na-K Co-transporter<br>Na-K Pump<br>K-Cl Cotransporter 2<br>Leak<br>Internal Ions
dNAC	Leak<br>Internal Ions	D-type Ka<br>Persistent Na<br>Transient K<br>Persistent K<br>Kv3.1<br>SKCa<br>HVA Ca<br>LVA Ca<br>Na-K Co-transporter<br>Na-K Pump<br>Leak<br>Internal Ions	D-type K<br>Transient K<br>Persistent K<br>Kv3.1<br>SKCa<br>HVA Ca<br>LVA Ca<br>Hyperpolarisation activated<br>Na-K Co-transporter<br>Na-K Pump<br>K-Cl Cotransporter 2<br>Leak<br>Internal Ions		D-type K<br>HVA Ca<br>LVA Ca<br>Hyperpolarisation activated<br>Na-K Co-transporter<br>Na-K Pump<br>K-Cl Cotransporter 2<br>Leak<br>Internal Ions

e-type	Myelinated Axon	AIS	Soma	Apical Dendrite	Basal Dendrite
bIR<br>bSTUT<br>cIR<br>cSTUT	Leak<br>Internal Ions	Stochastic K<br>Persistent Na<br>Transient K<br>Persistent K<br>Kv3.1<br>SKCa<br>HVA Ca<br>LVA Ca<br>Na-K Co-transporter<br>Na-K Pump<br>Leak<br>Internal Ions	Stochastic K<br>Transient K<br>Persistent K<br>Kv3.1<br>SKCa<br>HVA Ca<br>LVA Ca<br>Hyperpolarisation activated<br>Na-K Co-transporter<br>Na-K Pump<br>K-Cl Cotransporter 2<br>Leak<br>Internal Ions		Stochastic K<br>HVA Ca<br>LVA Ca<br>Hyperpolarisation activated<br>Na-K Co-transporter<br>Na-K Pump<br>K-Cl Cotransporter 2<br>Leak<br>Internal Ions
dSTUT	Leak<br>Internal Ions	D-type K<br>Stochastic K<br>Persistent Na<br>Transient K<br>Persistent K<br>Kv3.1<br>SKCa<br>HVA Ca<br>LVA Ca<br>Na-K Co-transporter<br>Na-K Pump<br>Leak<br>Internal Ions	D-type K<br>Stochastic K<br>Transient K<br>Persistent K<br>Kv3.1<br>SKCa<br>HVA Ca<br>LVA Ca<br>Hyperpolarisation activated<br>Na-K Co-transporter<br>Na-K Pump<br>K-Cl Cotransporter 2<br>Leak<br>Internal Ions		D-type K<br>Stochastic K<br>HVA Ca<br>LVA Ca<br>Hyperpolarisation activated<br>Na-K Co-transporter<br>Na-K Pump<br>K-Cl Cotransporter 2<br>Leak<br>Internal Ions

Table D: mechanisms and their neuron model locations for different e-types (Part 2).

**Property**	**Value**
Temperature	34 °C
Specific Membrane Capacitance (cm), Soma	$1\mu F/cm^2$
Specific Membrane Capacitance (cm), Dendrites	$2\mu F/cm^2$
Specific Membrane Capacitance (cm), Myelinated Axon	$0.02\mu F/cm^2$
Axial Resistance	100 Ωcm
Initial intracellular P ($[P]_i$)<br>Initial extracellular P ($[P]_o$)	2.180 mM<br>0.0 mM
Initial intracellular ATP ($[ATP]_i$)<br>Initial extracellular ATP ($[ATP]_o$)	1.385 mM<br>0.0 mM
Initial intracellular ADP ($[ADP]_i$)<br>Initial extracellular ADP ($[ADP]_o$)	0.058 mM<br>0.0 mM
Initial intracellular Na ($[Na]_i$)<br>Initial extracellular Na ($[Na]_o$)	10.0 mM<br>140.0 mM
Initial intracellular Ca ($[Ca]_i$)<br>Initial extracellular Ca ($[Ca]_o$)	5.0×10^{-5} mM<br>1.500 mM
Initial intracellular Cl ($[Cl]_i$)<br>Initial extracellular Cl ($[Cl]_o$)	6.0 mM<br>130.0 mM
Initial intracellular K ($[K]_i$)<br>Initial extracellular K ($[K]_o$)	140.0 mM<br>5.0 mM

Table E: parameters of the electrical model used in single neuron models and optimization

References

1. Colbert CM, Pan E. Ion channel properties underlying axonal action potential initiation in pyramidal neurons. Nature Neuroscience. 2002;5(6):533–538. doi:10/cdtnrk.

2. Magistretti J, Alonso A. Biophysical Properties and Slow Voltage-Dependent Inactivation of a Sustained Sodium Current in Entorhinal Cortex Layer-II Principal Neurons. The Journal of General Physiology. 1999;114(4):491–509. doi:10.1085/jgp.114.4.491.

3. Hille B. The permeability of the sodium channel to metal cations in myelinated nerve. The Journal of general physiology. 1972;59(6):637–658.

4. Korngreen A, Sakmann B. Voltage-gated K+ channels in layer 5 neocortical pyramidal neurones from young rats: subtypes and gradients. The Journal of Physiology. 2000;525(Pt 3):621. doi:10/b9hpsh.

5. Rettig J, Wunder F, Stocker M, Lichtinghagen R, Mastiaux F, Beckh S, et al. Characterization of a Shaw-related potassium channel family in rat brain. The EMBO Journal. 1992;11(7):2473–2486.

6. Grupe A, Schröter KH, Ruppersberg JP, Stocker M, Drewes T, Beckh S, et al. Cloning and expression of a human voltage-gated potassium channel. A novel member of the RCK potassium channel family. The EMBO Journal. 1990;9(6):1749–1756. doi:10/gjms8t.

7. Shu Y, Yu Y, Yang J, McCormick DA. Selective control of cortical axonal spikes by a slowly inactivating K^+ current. Proceedings of the National Academy of Sciences. 2007;104(27):11453–11458. doi:10.1073/pnas.0702041104.

8. Diba K, Koch C, Segev I. Spike propagation in dendrites with stochastic ion channels. Journal of Computational Neuroscience. 2006;20(1):77–84. doi:10/bmxqnr.

9. Mendonça PR, Vargas-Caballero M, Erdélyi F, Szabó G, Paulsen O, Robinson HP. Stochastic and deterministic dynamics of intrinsically irregular firing in cortical inhibitory interneurons. eLife. 2016;5:e16475. doi:10/f9rn9b.

10. Kohler M, Hirschberg B, Bond CT, Kinzie JM, Marrion NV, Maylie J, et al. Small-Conductance, Calcium-Activated Potassium Channels from Mammalian Brain. Science. 1996;273(5282):1709–1714. doi:10/bbmhfb.

11. Reuveni I, Friedman A, Amitai Y, Gutnick MJ. Stepwise repolarization from Ca2+ plateaus in neocortical pyramidal cells: evidence for nonhomogeneous distribution of HVA Ca2+ channels in dendrites. Journal of Neuroscience. 1993;13(11):4609–4621. doi:10/ghr972.

12. Sayer RJ, Schwindt PC, Crill WE. High- and low-threshold calcium currents in neurons acutely isolated from rat sensorimotor cortex. Neuroscience Letters. 1990;120(2):175–178. doi:10.1016/0304-3940(90)90031-4.

13. Dichter MA, Zona C. Calcium currents in cultured rat cortical neurons. Brain Research. 1989;492(1):219–229. doi:10/c792mn.

14. Avery RB, Johnston D. Multiple Channel Types Contribute to the Low-Voltage-Activated Calcium Current in Hippocampal CA3 Pyramidal Neurons. Journal of Neuroscience. 1996;16(18):5567–5582. doi:10/gjmtck.

15. Randall AD, Tsien RW. Contrasting biophysical and pharmacological properties of T-type and R-type calcium channels. Neuropharmacology. 1997;36(7):879–893. doi:10/b2fppd.

16. Kole MHP, Hallermann S, Stuart GJ. Single I_h Channels in Pyramidal Neuron Dendrites: Properties, Distribution, and Impact on Action Potential Output. The Journal of Neuroscience. 2006;26(6):1677–1687. doi:10.1523/JNEUROSCI.3664-05.2006.

17. Solomon J, Nerbonne J. Hyperpolarization-activated currents in isolated superior colliculus-projecting neurons from rat visual cortex. The Journal of physiology. 1993;462(1):393–420.

18. Hodgkin AL, Katz B. The effect of sodium ions on the electrical activity of the giant axon of the squid. The Journal of physiology. 1949;108(1):37.

19. Chapman JB, Johnson EA, Kootsey JM. Electrical and biochemical properties of an enzyme model of the sodium pump. The Journal of membrane biology. 1983;74:139–153.

20. Gentiletti D, Suffczynski P, Gnatkovsky V, de Curtis M. Changes of ionic concentrations during seizure transitions—a modeling study. International journal of neural systems. 2017;27(04):1750004.
