# Supplementary material for: A multiscale electro-metabolic model of a rat neocortical circuit reveals the impact of ageing on central cortical layers
Source: PLoS Comput Biol. 2025 May 20;21(5):e1013070. doi: 10.1371/journal.pcbi.1013070 (PMC12112163; doi:10.1371/journal.pcbi.1013070)
Supplement: S2 Text — Tables A–H: Julia ODEs. Tables I–X: Julia Fluxes. Tables Y–AB: Initial value of metabolism variables. (PDF) [file pcbi.1013070.s003.pdf]

S2 Text: A multiscale electro-metabolic model of a rat neocortical circuit reveals the impact of ageing on central cortical layers

Sofia Farina[1], Alessandro Cattabiani[1], Darshan Mandge[1], Polina Shichkova[1,2], James B. Isbister[1], Jean Jacquemier[1], Jim G. King[1], Henry Markram[1,3], Daniel Keller[1]

ODE	Expression
du[1]	0
du[2]	0.5*T2Jcorrection*(1000*(notBigg_J_KH_n(u_h_m_n,u_h_i_n,u_k_m_n)+ notBigg_J_K_n(u_k_m_n,u_notBigg_MitoMembrPotent_m_n))/W_x)
du[3]	0.5*T2Jcorrection*(1000*(-notBigg_J_MgATPx_n(u_notBigg_ATP_mx_m_n,u_mg2_m_n)- notBigg_J_MgADPx_n(u_mg2_m_n,u_notBigg_ADP_mx_m_n))/W_x)
du[4]	6.96*(notBigg_vMitoinn(u_pyr_m_n,u_nadh_m_n)+notBigg_vShuttlen(u_nadh_m_n,u_nadh_c_n)- notBigg_vMitooutn_n(u_nadh_m_n,u_nad_m_n,u_o2_c_n,u_adp_i_n,u_atp_i_n))
du[5]	0.5*T2Jcorrection*(1000*(+NADH2_u10mi_n(u_nadh_m_n,u_q10h2_m_n)- CYOR_u10mi_n(u_focytC_m_n,u_notBigg_MitoMembrPotent_m_n,u_q10h2_m_n,u_pi_m_n))/W_x)
du[6]	0.5*T2Jcorrection*(1000*(+2* CYOR_u10mi_n(u_focytC_m_n,u_notBigg_MitoMembrPotent_m_n,u_q10h2_m_n,u_pi_m_n)-2* CYOOm2i_n(u_focytC_m_n,u_notBigg_MitoMembrPotent_m_n,u_notBigg_Ctot_m_n,u_o2_c_n))/ W_i)
du[7]	notBigg_JO2fromCap2n(u_o2_b_b,u_o2_c_n)-0.6* notBigg_vMitooutn_n(u_nadh_m_n,u_nad_m_n,u_o2_c_n,u_adp_i_n,u_atp_i_n)
du[8]	0.5*T2Jcorrection*(1000*(+ ATPS4mi_n(u_notBigg_ATP_mx_m_n,u_notBigg_ADP_mx_m_n,u_pi_m_n)- ATPtm_n(u_notBigg_MitoMembrPotent_m_n))/W_x)
du[9]	0.5*T2Jcorrection*(1000*(- ATPS4mi_n(u_notBigg_ATP_mx_m_n,u_notBigg_ADP_mx_m_n,u_pi_m_n)+ ATPtm_n(u_notBigg_MitoMembrPotent_m_n))/W_x)
du[10]	0.5*T2Jcorrection*(1000*(notBigg_J_MgATPx_n(u_notBigg_ATP_mx_m_n,u_mg2_m_n))/W_x)
du[11]	0.5*T2Jcorrection*(1000*(notBigg_J_MgADPx_n(u_mg2_m_n,u_notBigg_ADP_mx_m_n))/W_x)
du[12]	0.5*T2Jcorrection*(1000*(- ATPS4mi_n(u_notBigg_ATP_mx_m_n,u_notBigg_ADP_mx_m_n,u_pi_m_n)+ notBigg_J_Pi1_n(u_h_m_n,u_h_i_n))/W_x)
du[13]	0.5*T2Jcorrection*(1000*(+notBigg_J_ATP_n(u_atp_c_n,u_atp_i_n)+ ATPtm_n(u_notBigg_MitoMembrPotent_m_n)+ADK1m_n(u_amp_i_n,u_atp_i_n,u_adp_i_n))/W_i)
du[14]	0.5*T2Jcorrection*(1000*(+notBigg_J_ADP_n(u_adp_i_n,u_adp_c_n)- ATPtm_n(u_notBigg_MitoMembrPotent_m_n)-2*ADK1m_n(u_amp_i_n,u_atp_i_n,u_adp_i_n))/ W_i)
du[15]	0.5*T2Jcorrection*(1000*(+notBigg_J_AMP_n(u_amp_i_n,u_amp_c_n)+ ADK1m_n(u_amp_i_n,u_atp_i_n,u_adp_i_n))/W_i)
du[16]	0.5*T2Jcorrection*(1000*(notBigg_J_MgATPi_n(u_notBigg_ATP_mi_i_n))/W_i)
du[17]	0.5*T2Jcorrection*(1000*(notBigg_J_MgADPi_n(u_notBigg_ADP_mi_i_n))/W_i)
du[18]	0.5*T2Jcorrection*(1000*(-notBigg_J_Pi1_n(u_h_m_n,u_h_i_n)+ notBigg_J_Pi2_n(u_pi_i_n,u_pi_c_n))/W_i)
du[19]	0.5*T2Jcorrection*(4*NADH2_u10mi_n(u_nadh_m_n,u_q10h2_m_n)+2* CYOR_u10mi_n(u_focytC_m_n,u_notBigg_MitoMembrPotent_m_n,u_q10h2_m_n,u_pi_m_n)+4* CYOOm2i_n(u_focytC_m_n,u_notBigg_MitoMembrPotent_m_n,u_notBigg_Ctot_m_n,u_o2_c_n)- n_A*ATPS4mi_n(u_notBigg_ATP_mx_m_n,u_notBigg_ADP_mx_m_n,u_pi_m_n)- ATPtm_n(u_notBigg_MitoMembrPotent_m_n)- notBigg_J_Hle_n(u_h_m_n,u_h_i_n,u_notBigg_MitoMembrPotent_m_n)- notBigg_J_K_n(u_k_m_n,u_notBigg_MitoMembrPotent_m_n))/CIM

Table A. Julia ODEs (Part 1)

ODE	Expression
du[20]	0
du[21]	0
du[22]	0
du[23]	(CKc_n(u_pcreat_c_n,u_atp_c_n,u_adp_c_n)+0.5*(1/6.96)*1000*(-notBigg_J_ATP_n(u_atp_c_n,u_atp_i_n))-HEX1_n(u_glc_D_c_n,u_atp_c_n,u_g6p_c_n)-PFK_n(u_f6p_c_n,u_atp_c_n)+PGK_n(u_13dpg_c_n,u_3pg_c_n,u_atp_c_n,u_adp_c_n)+PYK_n(u_pep_c_n,u_atp_c_n,u_adp_c_n))/(1-dAMPdATPn)
du[24]	0
du[25]	T2Jcorrection*(0.5*1000*r0509_n(u_nadh_m_n,u_pi_m_n)/W_x-FUMm_n(u_fum_m_n,u_mal_L_m_n))
du[26]	T2Jcorrection*(FUMm_n(u_fum_m_n,u_mal_L_m_n)-MDHm_n(u_nadh_m_n,u_oaa_m_n,u_nad_m_n,u_mal_L_m_n))+6.96*AKGMALtm_n(u_mal_L_c_n,u_akg_m_n,u_akg_c_n,u_mal_L_m_n)
du[27]	T2Jcorrection*(MDHm_n(u_nadh_m_n,u_oaa_m_n,u_nad_m_n,u_mal_L_m_n)-CSm_n(u_accoa_m_n,u_oaa_m_n,u_cit_m_n,u_coa_m_n))+ASPTAm_n(u_oaa_m_n,u_akg_m_n,u_glu_L_m_n,u_asp_L_m_n)
du[28]	T2Jcorrection*(0.5*SUCOASm_n(u_coa_m_n,u_succ_m_n,u_pi_m_n,u_adp_m_n,u_succoa_m_n,u_atp_m_n)-0.5*1000*r0509_n(u_nadh_m_n,u_pi_m_n)/W_x)+0.5*T2Jcorrection*OCOAT1m_n(u_acac_c_n,u_aacoa_m_n,u_succoa_m_n,u_succ_m_n)
du[29]	0.5*T2Jcorrection*(AKGDm_n(u_coa_m_n,u_nadh_m_n,u_ca2_m_n,u_adp_m_n,u_succoa_m_n,u_nad_m_n,u_atp_m_n,u_akg_m_n)-SUCOASm_n(u_coa_m_n,u_succ_m_n,u_pi_m_n,u_adp_m_n,u_succoa_m_n,u_atp_m_n))-0.5*T2Jcorrection*OCOAT1m_n(u_acac_c_n,u_aacoa_m_n,u_succoa_m_n,u_succ_m_n)
du[30]	T2Jcorrection*(CSm_n(u_accoa_m_n,u_oaa_m_n,u_cit_m_n,u_coa_m_n)-PDHm_n(u_pyr_m_n,u_coa_m_n,u_nad_m_n)-0.5*AKGDm_n(u_coa_m_n,u_nadh_m_n,u_ca2_m_n,u_adp_m_n,u_succoa_m_n,u_nad_m_n,u_atp_m_n,u_akg_m_n)+0.5*SUCOASm_n(u_coa_m_n,u_succ_m_n,u_pi_m_n,u_adp_m_n,u_succoa_m_n,u_atp_m_n)-0.5*ACACT1rm_n(u_aacoa_m_n,u_coa_m_n))
du[31]	0.5*T2Jcorrection*(ICDHxm_n(u_nadh_m_n,u_icit_m_n,u_nad_m_n)-AKGDm_n(u_coa_m_n,u_nadh_m_n,u_ca2_m_n,u_adp_m_n,u_succoa_m_n,u_nad_m_n,u_atp_m_n,u_akg_m_n))-ASPTAm_n(u_oaa_m_n,u_akg_m_n,u_glu_L_m_n,u_asp_L_m_n)-AKGMALtm_n(u_mal_L_c_n,u_akg_m_n,u_akg_c_n,u_mal_L_m_n)
du[32]	0
du[33]	0.5*T2Jcorrection*(ACONTm_n(u_icit_m_n,u_cit_m_n)-ICDHxm_n(u_nadh_m_n,u_icit_m_n,u_nad_m_n))
du[34]	T2Jcorrection*(CSm_n(u_accoa_m_n,u_oaa_m_n,u_cit_m_n,u_coa_m_n)-0.5*ACONTm_n(u_icit_m_n,u_cit_m_n))
du[35]	T2Jcorrection*(PDHm_n(u_pyr_m_n,u_coa_m_n,u_nad_m_n)-CSm_n(u_accoa_m_n,u_oaa_m_n,u_cit_m_n,u_coa_m_n))+T2Jcorrection*ACACT1rm_n(u_aacoa_m_n,u_coa_m_n)
du[36]	0.5*T2Jcorrection*(BDHm_n(u_acac_c_n,u_bhb_c_n,u_nadh_m_n,u_nad_m_n)-OCOAT1m_n(u_acac_c_n,u_aacoa_m_n,u_succoa_m_n,u_succ_m_n))
du[37]	0.5*T2Jcorrection*(OCOAT1m_n(u_acac_c_n,u_aacoa_m_n,u_succoa_m_n,u_succ_m_n)-ACACT1rm_n(u_aacoa_m_n,u_coa_m_n))
du[38]	6.96*PYRt2m_n(u_h_m_n,u_pyr_m_n,u_h_c_n,u_pyr_c_n)-T2Jcorrection*PDHm_n(u_pyr_m_n,u_coa_m_n,u_nad_m_n)
du[39]	0.44*BHBt_n(u_bhb_c_n,u_bhb_e_e)-BDHm_n(u_acac_c_n,u_bhb_c_n,u_nadh_m_n,u_nad_m_n)
du[40]	0.0275*notBigg_MCT1_bHB_b(u_bhb_e_e,u_bhb_b_b)-BHBt_n(u_bhb_c_n,u_bhb_e_e)-BHBt_a(u_bhb_c_a,u_bhb_e_e)
du[41]	0
du[42]	notBigg_JbHBTrArtCap(t,u_bhb_b_b)-notBigg_MCT1_bHB_b(u_bhb_e_e,u_bhb_b_b)
du[43]	0
du[44]	0

Table B. Julia ODEs (Part 2)

ODE	Expression
du[45]	0.5*T2Jcorrection*(ASPTAm_n(u_oaa_m_n,u_akg_m_n,u_glu_L_m_n,u_asp_L_m_n)+6.96*ASPGLUm_n(u_h_c_n,u_glu_L_c_n,u_asp_L_m_n,u_asp_L_c_n,u_notBigg_MitoMembrPotent_m_n,u_glu_L_m_n,u_h_m_n)+GLUNm_n(u_gln_L_c_n,u_glu_L_m_n))
du[46]	0
du[47]	0
du[48]	0
du[49]	ASPTA_n(u_glu_L_c_n,u_asp_L_c_n,u_oaa_c_n,u_akg_c_n)-ASPGLUm_n(u_h_c_n,u_glu_L_c_n,u_asp_L_m_n,u_asp_L_c_n,u_notBigg_MitoMembrPotent_m_n,u_glu_L_m_n,u_h_m_n)
du[50]	GAPD_n(u_nadh_c_n,u_nad_c_n,u_g3p_c_n,u_pi_c_n,u_13dpg_c_n)-LDH_L_n(u_nad_c_n,u_lac_L_c_n,u_nadh_c_n,u_pyr_c_n)-notBigg_vShuttlen(u_nadh_m_n,u_nadh_c_n)
du[51]	0
du[52]	0.5*T2Jcorrection*(1000*(notBigg_J_KH_a(u_h_i_a,u_h_m_a,u_k_m_a)+notBigg_J_K_a(u_k_m_a,u_notBigg_MitoMembrPotent_m_a))/W_x)
du[53]	0.5*T2Jcorrection*(1000*(-notBigg_J_MgATPx_a(u_notBigg_ATP_mx_m_a,u_mg2_m_a)-notBigg_J_MgADPx_a(u_notBigg_ADP_mx_m_a,u_mg2_m_a))/W_x)
du[54]	6.96*(notBigg_vMitoing(u_nadh_m_a,u_pyr_m_a)+notBigg_vShuttleg(u_nadh_c_a,u_nadh_m_a)-notBigg_vMitooutg_a(u_atp_i_a,u_nadh_m_a,u_nad_m_a,u_o2_c_a,u_adp_i_a))
du[55]	0.5*T2Jcorrection*(1000*(+NADH2_u10mi_a(u_nadh_m_a,u_q10h2_m_a)-CYOR_u10mi_a(u_focytC_m_a,u_q10h2_m_a,u_pi_m_a,u_notBigg_MitoMembrPotent_m_a))/W_x)
du[56]	0.5*T2Jcorrection*(1000*(+2*CYOR_u10mi_a(u_focytC_m_a,u_q10h2_m_a,u_pi_m_a,u_notBigg_MitoMembrPotent_m_a)-2*CYOOm2i_a(u_notBigg_Ctot_m_a,u_focytC_m_a,u_notBigg_MitoMembrPotent_m_a,u_o2_c_a))/W_i)
du[57]	notBigg_JO2fromCap2a(u_o2_b_b,u_o2_c_a)-0.6*notBigg_vMitooutg_a(u_atp_i_a,u_nadh_m_a,u_nad_m_a,u_o2_c_a,u_adp_i_a)
du[58]	0.5*T2Jcorrection*(1000*(+ATPS4mi_a(u_notBigg_ATP_mx_m_a,u_pi_m_a,u_notBigg_ADP_mx_m_a)-ATPtm_a(u_notBigg_MitoMembrPotent_m_a))/W_x)
du[59]	0.5*T2Jcorrection*(1000*(-ATPS4mi_a(u_notBigg_ATP_mx_m_a,u_pi_m_a,u_notBigg_ADP_mx_m_a)+ATPtm_a(u_notBigg_MitoMembrPotent_m_a))/W_x)
du[60]	0.5*T2Jcorrection*(1000*(notBigg_J_MgATPx_a(u_notBigg_ATP_mx_m_a,u_mg2_m_a))/W_x)
du[61]	0.5*T2Jcorrection*(1000*(notBigg_J_MgADPx_a(u_notBigg_ADP_mx_m_a,u_mg2_m_a))/W_x)
du[62]	0.5*T2Jcorrection*(1000*(-ATPS4mi_a(u_notBigg_ATP_mx_m_a,u_pi_m_a,u_notBigg_ADP_mx_m_a)+notBigg_J_Pi1_a(u_h_i_a,u_h_m_a))/W_x)
du[63]	0.5*T2Jcorrection*(1000*(+notBigg_J_ATP_a(u_atp_i_a,u_atp_c_a)+ATPtm_a(u_notBigg_MitoMembrPotent_m_a)+ADK1m_a(u_atp_i_a,u_adp_i_a,u_amp_i_a))/W_i)
du[64]	0.5*T2Jcorrection*(1000*(+notBigg_J_ADP_a(u_adp_c_a,u_adp_i_a)-ATPtm_a(u_notBigg_MitoMembrPotent_m_a)-2*ADK1m_a(u_atp_i_a,u_adp_i_a,u_amp_i_a))/W_i)
du[65]	0.5*T2Jcorrection*(1000*(+notBigg_J_AMP_a(u_amp_i_a,u_amp_c_a)+ADK1m_a(u_atp_i_a,u_adp_i_a,u_amp_i_a))/W_i)
du[66]	0.5*T2Jcorrection*(1000*(notBigg_J_MgATPi_a(u_notBigg_ATP_mi_i_a))/W_i)
du[67]	0.5*T2Jcorrection*(1000*(notBigg_J_MgADPi_a(u_notBigg_ADP_mi_i_a))/W_i)
du[68]	0.5*T2Jcorrection*(1000*(-notBigg_J_Pi1_a(u_h_i_a,u_h_m_a)+notBigg_J_Pi2_a(u_pi_i_a,u_pi_c_a))/W_i)
du[69]	0.5*T2Jcorrection*((4*NADH2_u10mi_a(u_nadh_m_a,u_q10h2_m_a)+2*CYOR_u10mi_a(u_focytC_m_a,u_q10h2_m_a,u_pi_m_a,u_notBigg_MitoMembrPotent_m_a)+4*CYOOm2i_a(u_notBigg_Ctot_m_a,u_focytC_m_a,u_notBigg_MitoMembrPotent_m_a,u_o2_c_a)-n_A*ATPS4mi_a(u_notBigg_ATP_mx_m_a,u_pi_m_a,u_notBigg_ADP_mx_m_a)-ATPtm_a(u_notBigg_MitoMembrPotent_m_a)-notBigg_J_Hle_a(u_h_i_a,u_h_m_a,u_notBigg_MitoMembrPotent_m_a)-notBigg_J_K_a(u_k_m_a,u_notBigg_MitoMembrPotent_m_a))/CIM)

Table C. Julia ODEs (Part 3)

ODE	Expression
du[70]	0
du[71]	0
du[72]	0
du[73]	(-(u_ca2_c_a/cai0_ca_ion)*(1+xNEmod*(u[178]/(KdNEmod+u[178])))* ADNCYC_a(u_camp_c_a,u_atp_c_a)+CKc_a(u_adp_c_a,u_atp_c_a,u_pcreat_c_a)+0.5*(1/6.96) *1000*(-notBigg_J_ATP_a(u_atp_i_a,u_atp_c_a))- HEX1_a(u_atp_c_a,u_g6p_c_a,u_glc_D_c_a)-PFK_a(u_atp_c_a,u_f26bp_c_a,u_f6p_c_a)- PFK26_a(u_adp_c_a,u_atp_c_a,u_f26bp_c_a,u_f6p_c_a)+ PGK_a(u_adp_c_a,u_atp_c_a,u_3pg_c_a,u_13dpg_c_a)+ PYK_a(u_adp_c_a,u_atp_c_a,u_pep_c_a)-0.15*(7/4)*vPumpg-vATPasesg)/(1-dAMPdATPg)
du[74]	0
du[75]	0.5*T2Jcorrection*(1000*r0509_a(u_nadh_m_a,u_pi_m_a)/W_x- FUMm_a(u_mal_L_m_a,u_fum_m_a))
du[76]	0.5*T2Jcorrection*(FUMm_a(u_mal_L_m_a,u_fum_m_a)- MDHm_a(u_nad_m_a,u_oaa_m_a,u_mal_L_m_a,u_nadh_m_a))
du[77]	0.5*T2Jcorrection*(MDHm_a(u_nad_m_a,u_oaa_m_a,u_mal_L_m_a,u_nadh_m_a)- CSm_a(u_oaa_m_a,u_cit_m_a,u_accoa_m_a,u_coa_m_a)+ PCm_a(u_co2_m_a,u_oaa_m_a,u_pyr_m_a,u_atp_m_a,u_adp_m_a))
du[78]	0.5*T2Jcorrection* (SUCOASm_a(u_coa_m_a,u_succoa_m_a,u_atp_m_a,u_succ_m_a,u_pi_m_a,u_adp_m_a)-1000* r0509_a(u_nadh_m_a,u_pi_m_a)/W_x)
du[79]	0.5*T2Jcorrection* (AKGDm_a(u_coa_m_a,u_nadh_m_a,u_akg_m_a,u_ca2_m_a,u_nad_m_a,u_succoa_m_a,u_atp_ m_a,u_adp_m_a)- SUCOASm_a(u_coa_m_a,u_succoa_m_a,u_atp_m_a,u_succ_m_a,u_pi_m_a,u_adp_m_a))
du[80]	0.5*T2Jcorrection*(CSm_a(u_oaa_m_a,u_cit_m_a,u_accoa_m_a,u_coa_m_a)- PDHm_a(u_nad_m_a,u_pyr_m_a,u_coa_m_a)- AKGDm_a(u_coa_m_a,u_nadh_m_a,u_akg_m_a,u_ca2_m_a,u_nad_m_a,u_succoa_m_a,u_atp_m _a,u_adp_m_a)+ SUCOASm_a(u_coa_m_a,u_succoa_m_a,u_atp_m_a,u_succ_m_a,u_pi_m_a,u_adp_m_a))
du[81]	0.5*T2Jcorrection*(ICDHxm_a(u_nad_m_a,u_nadh_m_a,u_icit_m_a)- AKGDm_a(u_coa_m_a,u_nadh_m_a,u_akg_m_a,u_ca2_m_a,u_nad_m_a,u_succoa_m_a,u_atp_m _a,u_adp_m_a)+GLUDxm_a(u_nad_m_a,u_nadh_m_a,u_glu_L_c_a,u_akg_m_a))
du[82]	0
du[83]	0.5*T2Jcorrection*(ACONTm_a(u_cit_m_a,u_icit_m_a)- ICDHxm_a(u_nad_m_a,u_nadh_m_a,u_icit_m_a))
du[84]	0.5*T2Jcorrection*(CSm_a(u_oaa_m_a,u_cit_m_a,u_accoa_m_a,u_coa_m_a)- ACONTm_a(u_cit_m_a,u_icit_m_a))
du[85]	0.5*T2Jcorrection*(PDHm_a(u_nad_m_a,u_pyr_m_a,u_coa_m_a)- CSm_a(u_oaa_m_a,u_cit_m_a,u_accoa_m_a,u_coa_m_a))
du[86]	0
du[87]	0
du[88]	6.96*PYRt2m_a(u_h_m_a,u_pyr_c_a,u_h_c_a,u_pyr_m_a)-T2Jcorrection* (PDHm_a(u_nad_m_a,u_pyr_m_a,u_coa_m_a)+ PCm_a(u_co2_m_a,u_oaa_m_a,u_pyr_m_a,u_atp_m_a,u_adp_m_a))
du[89]	T2Jcorrection*(GLNt4_n(u_gln_L_c_n,u_gln_L_e_e)-GLUNm_n(u_gln_L_c_n,u_glu_L_m_n))
du[90]	T2Jcorrection*(-GLNt4_n(u_gln_L_c_n,u_gln_L_e_e)+GLNt4_a(u_gln_L_c_a,u_gln_L_e_e))
du[91]	T2Jcorrection*(-GLNt4_a(u_gln_L_c_a,u_gln_L_e_e)+ GLNS_a(u_adp_c_a,u_atp_c_a,u_glu_L_c_a))
du[92]	T2Jcorrection*(0.0266* GLUt6_a(u_na1_c_a,u_notBigg_Va_c_a,u_k_e_e,u_glu_L_syn_syn,u_k_c_a,u_glu_L_c_a)-GLNS_a(u_adp_c_a,u_atp_c_a,u_glu_L_c_a)- GLUDxm_a(u_nad_m_a,u_nadh_m_a,u_glu_L_c_a,u_akg_m_a))
du[93]	0
du[94]	0
du[95]	0
du[96]	0
du[97]	0
du[98]	0

Table D. Julia ODEs (Part 4)

ODE	Expression
du[99]	0
du[100]	0
du[101]	0
du[102]	0
du[103]	0
du[104]	0
du[105]	0
du[106]	0
du[107]	0
du[108]	0
du[109]	0
du[110]	0
du[111]	0
du[112]	0
du[113]	notBigg_JdHbin(t,u_o2_b_b)-notBigg_JdHbout(t,u_notBigg_ddHb_b_b)
du[114]	notBigg_JO2art2cap(t,u_o2_b_b)-(eto_n/eto_b)*notBigg_JO2fromCap2n(u_o2_b_b,u_o2_c_n)-(eto_a/eto_b)*notBigg_JO2fromCap2a(u_o2_b_b,u_o2_c_a)
du[115]	notBigg_trGLC_art_cap(t,u_glc_D_b_b)-notBigg_JGlc_be(u_glc_D_ecsEndothelium_ecsEndothelium,u_glc_D_b_b)
du[116]	0.32*notBigg_JGlc_be(u_glc_D_ecsEndothelium_ecsEndothelium,u_glc_D_b_b)-notBigg_JGlc_e2ecsBA(u_glc_D_ecsBA_ecsBA,u_glc_D_ecsEndothelium_ecsEndothelium)
du[117]	1.13*notBigg_JGlc_e2ecsBA(u_glc_D_ecsBA_ecsBA,u_glc_D_ecsEndothelium_ecsEndothelium)-notBigg_JGlc_ecsBA2a(u_glc_D_ecsBA_ecsBA,u_glc_D_c_a)-notBigg_JGlc_diffEcs(u_glc_D_ecsBA_ecsBA,u_glc_D_ecsAN_ecsAN)
du[118]	0.06*notBigg_JGlc_ecsBA2a(u_glc_D_ecsBA_ecsBA,u_glc_D_c_a)-HEX1_a(u_atp_c_a,u_g6p_c_a,u_glc_D_c_a)-notBigg_JGlc_a2ecsAN(u_glc_D_ecsAN_ecsAN,u_glc_D_c_a)
du[119]	1.35*notBigg_JGlc_a2ecsAN(u_glc_D_ecsAN_ecsAN,u_glc_D_c_a)-notBigg_JGlc_ecsAN2n(u_glc_D_c_n,u_glc_D_ecsAN_ecsAN)+0.08*notBigg_JGlc_diffEcs(u_glc_D_ecsBA_ecsBA,u_glc_D_ecsAN_ecsAN)
du[120]	0.41*notBigg_JGlc_ecsAN2n(u_glc_D_c_n,u_glc_D_ecsAN_ecsAN)-HEX1_n(u_glc_D_c_n,u_atp_c_n,u_g6p_c_n)
du[121]	HEX1_n(u_glc_D_c_n,u_atp_c_n,u_g6p_c_n)-PGI_n(u_f6p_c_n,u_g6p_c_n)-G6PDH2r_n(u_g6p_c_n,u_nadph_c_n,u_nadp_c_n,u_6pgl_c_n)
du[122]	HEX1_a(u_atp_c_a,u_g6p_c_a,u_glc_D_c_a)-PGI_a(u_g6p_c_a,u_f6p_c_a)-G6PDH2r_a(u_6pgl_c_a,u_nadp_c_a,u_g6p_c_a,u_nadph_c_a)+PGMT_a(u_g1p_c_a,u_g6p_c_a)
du[123]	PGI_n(u_f6p_c_n,u_g6p_c_n)-PFK_n(u_f6p_c_n,u_atp_c_n)-TKT2_n(u_f6p_c_n,u_e4p_c_n,u_xu5p_D_c_n,u_g3p_c_n)+TALA_n(u_f6p_c_n,u_e4p_c_n,u_g3p_c_n,u_s7p_c_n)
du[124]	PGI_a(u_g6p_c_a,u_f6p_c_a)-PFK_a(u_atp_c_a,u_f26bp_c_a,u_f6p_c_a)-PFK26_a(u_adp_c_a,u_atp_c_a,u_f26bp_c_a,u_f6p_c_a)-TKT2_a(u_g3p_c_a,u_e4p_c_a,u_xu5p_D_c_a,u_f6p_c_a)+TALA_a(u_g3p_c_a,u_e4p_c_a,u_s7p_c_a,u_f6p_c_a)
du[125]	PFK_n(u_f6p_c_n,u_atp_c_n)-FBA_n(u_fdp_c_n,u_dhap_c_n,u_g3p_c_n)

Table E. Julia ODEs (Part 5)

ODE	Expression
du[126]	PFK_a(u_atp_c_a,u_f26bp_c_a,u_f6p_c_a)-FBA_a(u_g3p_c_a,u_fdp_c_a,u_dhap_c_a)
du[127]	PFK26_a(u_adp_c_a,u_atp_c_a,u_f26bp_c_a,u_f6p_c_a)
du[128]	GLCS2_a(u_notBigg_GS_c_a,u_udpg_c_a)-<br>GLCP_a(u_glycogen_c_a,u_notBigg_GPa_c_a,u_camp_c_a,u_notBigg_GPb_c_a)
du[129]	0
du[130]	0
du[131]	10*GLCP_a(u_glycogen_c_a,u_notBigg_GPa_c_a,u_camp_c_a,u_notBigg_GPb_c_a)-<br>PGMT_a(u_g1p_c_a,u_g6p_c_a)-GALUi_a(u_ppi_c_a,u_g1p_c_a,u_udpg_c_a,u_utp_c_a)
du[132]	FBA_n(u_fdp_c_n,u_dhap_c_n,u_g3p_c_n)-<br>GAPD_n(u_nadh_c_n,u_nad_c_n,u_g3p_c_n,u_pi_c_n,u_13dpg_c_n)+<br>TPI_n(u_dhap_c_n,u_g3p_c_n)-TKT2_n(u_f6p_c_n,u_e4p_c_n,u_xu5p_D_c_n,u_g3p_c_n)-<br>TALA_n(u_f6p_c_n,u_e4p_c_n,u_g3p_c_n,u_s7p_c_n)+<br>TKT1_n(u_s7p_c_n,u_r5p_c_n,u_xu5p_D_c_n,u_g3p_c_n)
du[133]	FBA_a(u_g3p_c_a,u_fdp_c_a,u_dhap_c_a)-<br>GAPD_a(u_nadh_c_a,u_nad_c_a,u_pi_c_a,u_13dpg_c_a,u_g3p_c_a)+<br>TPI_a(u_g3p_c_a,u_dhap_c_a)-TKT2_a(u_g3p_c_a,u_e4p_c_a,u_xu5p_D_c_a,u_f6p_c_a)-<br>TALA_a(u_g3p_c_a,u_e4p_c_a,u_s7p_c_a,u_f6p_c_a)+<br>TKT1_a(u_r5p_c_a,u_g3p_c_a,u_s7p_c_a,u_xu5p_D_c_a)
du[134]	FBA_n(u_fdp_c_n,u_dhap_c_n,u_g3p_c_n)-TPI_n(u_dhap_c_n,u_g3p_c_n)
du[135]	FBA_a(u_g3p_c_a,u_fdp_c_a,u_dhap_c_a)-TPI_a(u_g3p_c_a,u_dhap_c_a)
du[136]	GAPD_n(u_nadh_c_n,u_nad_c_n,u_g3p_c_n,u_pi_c_n,u_13dpg_c_n)-<br>PGK_n(u_13dpg_c_n,u_3pg_c_n,u_atp_c_n,u_adp_c_n)
du[137]	GAPD_a(u_nadh_c_a,u_nad_c_a,u_pi_c_a,u_13dpg_c_a,u_g3p_c_a)-<br>PGK_a(u_adp_c_a,u_atp_c_a,u_3pg_c_a,u_13dpg_c_a)
du[138]	GAPD_a(u_nadh_c_a,u_nad_c_a,u_pi_c_a,u_13dpg_c_a,u_g3p_c_a)-<br>LDH_L_a(u_nad_c_a,u_nadh_c_a,u_lac_L_c_a,u_pyr_c_a)-<br>notBigg_vShuttleg(u_nadh_c_a,u_nadh_m_a)
du[139]	0
du[140]	0
du[141]	PGK_n(u_13dpg_c_n,u_3pg_c_n,u_atp_c_n,u_adp_c_n)-PGM_n(u_2pg_c_n,u_3pg_c_n)
du[142]	PGK_a(u_adp_c_a,u_atp_c_a,u_3pg_c_a,u_13dpg_c_a)-PGM_a(u_2pg_c_a,u_3pg_c_a)
du[143]	PGM_n(u_2pg_c_n,u_3pg_c_n)-ENO_n(u_pep_c_n,u_2pg_c_n)
du[144]	PGM_a(u_2pg_c_a,u_3pg_c_a)-ENO_a(u_2pg_c_a,u_pep_c_a)
du[145]	ENO_n(u_pep_c_n,u_2pg_c_n)-PYK_n(u_pep_c_n,u_atp_c_n,u_adp_c_n)
du[146]	ENO_a(u_2pg_c_a,u_pep_c_a)-PYK_a(u_adp_c_a,u_atp_c_a,u_pep_c_a)
du[147]	PYK_n(u_pep_c_n,u_atp_c_n,u_adp_c_n)-<br>PYRt2m_n(u_h_m_n,u_pyr_m_n,u_h_c_n,u_pyr_c_n)-<br>LDH_L_n(u_nad_c_n,u_lac_L_c_n,u_nadh_c_n,u_pyr_c_n)
du[148]	PYK_a(u_adp_c_a,u_atp_c_a,u_pep_c_a)-<br>PYRt2m_a(u_h_m_a,u_pyr_c_a,u_h_c_a,u_pyr_m_a)-<br>LDH_L_a(u_nad_c_a,u_nadh_c_a,u_lac_L_c_a,u_pyr_c_a)
du[149]	notBigg_JLacTr_b(u_lac_L_b_b,t)-notBigg_MCT1_LAC_b(u_lac_L_b_b,u_lac_L_e_e)-<br>notBigg_vLACgc(u_lac_L_b_b,u_lac_L_c_a)
du[150]	0.0275*notBigg_MCT1_LAC_b(u_lac_L_b_b,u_lac_L_e_e)-<br>L_LACt2r_a(u_lac_L_e_e,u_lac_L_c_a)-L_LACt2r_n(u_lac_L_e_e,u_lac_L_c_n)+<br>notBigg_jLacDiff_e(u_lac_L_e_e)

Table F. Julia ODEs (Part 6)

ODE	Expression
du[151]	0.8*L_LACt2r_a(u_lac_L_e_e,u_lac_L_c_a)+0.022* notBigg_vLACgc(u_lac_L_b_b,u_lac_L_c_a)+ LDH_L_a(u_nad_c_a,u_nadh_c_a,u_lac_L_c_a,u_pyr_c_a)
du[152]	0.44*L_LACt2r_n(u_lac_L_e_e,u_lac_L_c_n)+ LDH_L_n(u_nad_c_n,u_lac_L_c_n,u_nadh_c_n,u_pyr_c_n)
du[153]	G6PDH2r_n(u_g6p_c_n,u_nadph_c_n,u_nadp_c_n,u_6pgl_c_n)+ GND_n(u_6pgc_c_n,u_nadph_c_n,u_nadp_c_n,u_ru5p_D_c_n)- notBigg_psiNADPHox_n(u_nadph_c_n)-GTHO_n(u_nadph_c_n,u_gthox_c_n)
du[154]	G6PDH2r_a(u_6pgl_c_a,u_nadp_c_a,u_g6p_c_a,u_nadph_c_a)+ GND_a(u_6pgc_c_a,u_nadp_c_a,u_ru5p_D_c_a,u_nadph_c_a)- notBigg_psiNADPHox_a(u_nadph_c_a)-GTHO_a(u_gthox_c_a,u_nadph_c_a)
du[155]	G6PDH2r_n(u_g6p_c_n,u_nadph_c_n,u_nadp_c_n,u_6pgl_c_n)- PGL_n(u_6pgc_c_n,u_6pgl_c_n)
du[156]	G6PDH2r_a(u_6pgl_c_a,u_nadp_c_a,u_g6p_c_a,u_nadph_c_a)- PGL_a(u_6pgc_c_a,u_6pgl_c_a)
du[157]	PGL_n(u_6pgc_c_n,u_6pgl_c_n)- GND_n(u_6pgc_c_n,u_nadph_c_n,u_nadp_c_n,u_ru5p_D_c_n)
du[158]	PGL_a(u_6pgc_c_a,u_6pgl_c_a)- GND_a(u_6pgc_c_a,u_nadp_c_a,u_ru5p_D_c_a,u_nadph_c_a)
du[159]	GND_n(u_6pgc_c_n,u_nadph_c_n,u_nadp_c_n,u_ru5p_D_c_n)- RPE_n(u_xu5p_D_c_n,u_ru5p_D_c_n)-RPI_n(u_r5p_c_n,u_ru5p_D_c_n)
du[160]	GND_a(u_6pgc_c_a,u_nadp_c_a,u_ru5p_D_c_a,u_nadph_c_a)- RPE_a(u_ru5p_D_c_a,u_xu5p_D_c_a)-RPI_a(u_r5p_c_a,u_ru5p_D_c_a)
du[161]	RPI_n(u_r5p_c_n,u_ru5p_D_c_n)- TKT1_n(u_s7p_c_n,u_r5p_c_n,u_xu5p_D_c_n,u_g3p_c_n)
du[162]	RPI_a(u_r5p_c_a,u_ru5p_D_c_a)- TKT1_a(u_r5p_c_a,u_g3p_c_a,u_s7p_c_a,u_xu5p_D_c_a)
du[163]	RPE_n(u_xu5p_D_c_n,u_ru5p_D_c_n)- TKT1_n(u_s7p_c_n,u_r5p_c_n,u_xu5p_D_c_n,u_g3p_c_n)+ TKT2_n(u_f6p_c_n,u_e4p_c_n,u_xu5p_D_c_n,u_g3p_c_n)
du[164]	RPE_a(u_ru5p_D_c_a,u_xu5p_D_c_a)- TKT1_a(u_r5p_c_a,u_g3p_c_a,u_s7p_c_a,u_xu5p_D_c_a)+ TKT2_a(u_g3p_c_a,u_e4p_c_a,u_xu5p_D_c_a,u_f6p_c_a)
du[165]	TKT1_n(u_s7p_c_n,u_r5p_c_n,u_xu5p_D_c_n,u_g3p_c_n)- TALA_n(u_f6p_c_n,u_e4p_c_n,u_g3p_c_n,u_s7p_c_n)
du[166]	TKT1_a(u_r5p_c_a,u_g3p_c_a,u_s7p_c_a,u_xu5p_D_c_a)- TALA_a(u_g3p_c_a,u_e4p_c_a,u_s7p_c_a,u_f6p_c_a)
du[167]	TKT2_n(u_f6p_c_n,u_e4p_c_n,u_xu5p_D_c_n,u_g3p_c_n)+ TALA_n(u_f6p_c_n,u_e4p_c_n,u_g3p_c_n,u_s7p_c_n)
du[168]	TKT2_a(u_g3p_c_a,u_e4p_c_a,u_xu5p_D_c_a,u_f6p_c_a)+ TALA_a(u_g3p_c_a,u_e4p_c_a,u_s7p_c_a,u_f6p_c_a)
du[169]	2*(GTHO_n(u_nadph_c_n,u_gthox_c_n)-GTHP_n(u_gthrd_c_n))+GTHS_n(u_gthrd_c_n)
du[170]	2*(GTHO_a(u_gthox_c_a,u_nadph_c_a)-GTHP_a(u_gthrd_c_a))+GTHS_a(u_gthrd_c_a)
du[171]	-GTHO_n(u_nadph_c_n,u_gthox_c_n)+GTHP_n(u_gthrd_c_n)
du[172]	-GTHO_a(u_gthox_c_a,u_nadph_c_a)+GTHP_a(u_gthrd_c_a)
du[173]	0
du[174]	-CKc_n(u_pcreat_c_n,u_atp_c_n,u_adp_c_n)
du[175]	0

Table G. Julia ODEs (Part 7)

ODE	Expression
du[176]	-CKc_a(u_adp_c_a,u_atp_c_a,u_pcreat_c_a)
du[177]	(u_ca2_c_a/cai0_ca_ion)*(1+xNEmod*(u[178]/(KdNEmod+u[178])))* ADNCYC_a(u_camp_c_a,u_atp_c_a)-PDE1_a(u_camp_c_a)
du[178]	0
du[179]	0
du[180]	0
du[181]	0
du[182]	notBigg_psiPHK_a(u_ca2_c_a,u_glycogen_c_a,u_g1p_c_a,u_notBigg_PHKa_c_a,u_notBigg_GPa_c_a,u_udpg_c_a)
du[183]	- notBigg_psiPHK_a(u_ca2_c_a,u_glycogen_c_a,u_g1p_c_a,u_notBigg_PHKa_c_a,u_notBigg_GPa_c_a,u_udpg_c_a)

Table H. Julia ODEs (Part 8)

Fluxes
r0509_n(u_nadh_m_n,u_pi_m_n) = 1*(mito_scale * x_DH*(r_DH*NAD_x_n-(1e-3*u_nadh_m_n)) *((1+(1e-3*u_pi_m_n)/k_Pi1)/(1+(1e-3*u_pi_m_n)/k_Pi2)))
NADH2_u10mi_n(u_nadh_m_n,u_q10h2_m_n) = 1*(mito_scale *x_C1*(exp(-(dG_C1op_n+4*dG_H_n)/etcRT)*(1e-3*u_nadh_m_n)*Q_n-NAD_x_n*(1e-3*u_q10h2_m_n)))
CYOR_u10mi_n(u_focytC_m_n,u_notBigg_MitoMembrPotent_m_n,u_q10h2_m_n,u_pi_m_n) = 1 *(mito_scale *x_C3*((1+(1e-3*u_pi_m_n)/k_Pi3)/(1+(1e-3*u_pi_m_n)/k_Pi4))*(exp(-(dG_C3op_n+4*dG_H_n-2*etcF*u_notBigg_MitoMembrPotent_m_n)/(2*etcRT))*Cox_n*(1e-3 *u_q10h2_m_n)0̂.5-(1e-3*u_focytC_m_n)*Q_n0̂.5))
CYOOm2i_n(u_focytC_m_n,u_notBigg_MitoMembrPotent_m_n,u_notBigg_Ctot_m_n,u_o2_c_n) = 1*(mito_scale *x_C4*((1e-3*u_o2_c_n)/((1e-3*u_o2_c_n)+k_O2))*((1e-3*u_focytC_m_n) /(1e-3*u_notBigg_Ctot_m_n))*(exp(-(dG_C4op_n+2*dG_H_n)/(2*etcRT))*(1e-3*u_focytC_m_n) *((1e-3*u_o2_c_n)0̂.25)-Cox_n*exp(etcF*u_notBigg_MitoMembrPotent_m_n/etcRT)))
ATPS4mi_n(u_notBigg_ATP_mx_m_n,u_notBigg_ADP_mx_m_n,u_pi_m_n) = 1*(mito_scale * x_F1*(exp(-(dG_F1op_n-n_A*dG_H_n)/etcRT)*(K_DD/K_DT)*(1e-3*u_notBigg_ADP_mx_m_n)*(1e -3*u_pi_m_n)-(1e-3*u_notBigg_ATP_mx_m_n)))
ATPtm_n(u_notBigg_MitoMembrPotent_m_n) = 1*mito_scale * (x_ANT*(ADP_fi_n/(ADP_fi_n +ATP_fi_n*exp(-etcF*(0.35*u_notBigg_MitoMembrPotent_m_n)/etcRT))-ADP_fx_n/(ADP_fx_n +ATP_fx_n*exp(-etcF*(-0.65*u_notBigg_MitoMembrPotent_m_n)/etcRT)))*(ADP_fi_n/(ADP_fi_n +k_mADP)))
notBigg_J_Pi1_n(u_h_m_n,u_h_i_n) = 1*mito_scale * (x_Pi1*((1e-3*u_h_m_n)*H2PIi_n-(1e -3*u_h_i_n)*H2PIx_n)/(H2PIi_n+k_PiH))
notBigg_J_Hle_n(u_h_m_n,u_h_i_n,u_notBigg_MitoMembrPotent_m_n) = 1*mito_scale * (x_Hle*u_notBigg_MitoMembrPotent_m_n*((1e-3*u_h_i_n)*exp(etcF *u_notBigg_MitoMembrPotent_m_n/etcRT)-(1e-3*u_h_m_n))/(exp(etcF *u_notBigg_MitoMembrPotent_m_n/etcRT)-1))
notBigg_J_KH_n(u_h_m_n,u_h_i_n,u_k_m_n) = 1*mito_scale * (x_KH*(K_i*(1e-3*u_h_m_n)-(1e-3*u_k_m_n)*(1e-3*u_h_i_n)))
notBigg_J_K_n(u_k_m_n,u_notBigg_MitoMembrPotent_m_n) = 1*mito_scale * (x_K *u_notBigg_MitoMembrPotent_m_n*(K_i*exp(etcF*u_notBigg_MitoMembrPotent_m_n/etcRT) -(1e-3*u_k_m_n))/(exp(etcF*u_notBigg_MitoMembrPotent_m_n/etcRT)-1))

Table I. Julia Fluxes (Part 1)

Fluxes
ADK1m_n(u_amp_i_n,u_atp_i_n,u_adp_i_n) = 1*mito_scale * (x_AK*(K_AK*(1e-3*u_adp_i_n)*(1e-3*u_adp_i_n)-(1e-3*u_amp_i_n)*(1e-3*u_atp_i_n)))
notBigg_J_AMP_n(u_amp_i_n,u_amp_c_n) = 1*mito_scale * (gamma*x_A*((1e-3*u_amp_c_n) -(1e-3*u_amp_i_n)))
notBigg_J_ADP_n(u_adp_i_n,u_adp_c_n) = 1*mito_scale * (gamma*x_A*((1e-3*u_adp_c_n) -(1e-3*u_adp_i_n)))
notBigg_J_ATP_n(u_atp_c_n,u_atp_i_n) = 1*mito_scale * (gamma*x_A*((1e-3*u_atp_c_n) -(1e-3*u_atp_i_n)))
notBigg_J_Pi2_n(u_pi_i_n,u_pi_c_n) = 1*mito_scale * (gamma*x_Pi2*(1e-3*u_pi_c_n-(1e-3 *u_pi_i_n)))
notBigg_J_Ht_n(u_h_i_n,u_h_c_n) = 1*mito_scale * (gamma*x_Ht*(1e-3*u_h_c_n-(1e-3 *u_h_i_n)))
notBigg_J_MgATPx_n(u_notBigg_ATP_mx_m_n,u_mg2_m_n) = 1*mito_scale * (x_MgA*(ATP_fx_n *(1e-3*u_mg2_m_n)-K_DT*(1e-3*u_notBigg_ATP_mx_m_n)))
notBigg_J_MgADPx_n(u_mg2_m_n,u_notBigg_ADP_mx_m_n) = 1*mito_scale * (x_MgA*(ADP_fx_n *(1e-3*u_mg2_m_n)-K_DD*(1e-3*u_notBigg_ADP_mx_m_n)))
notBigg_J_MgATPi_n(u_notBigg_ATP_mi_i_n) = 1*mito_scale * (x_MgA*(ATP_fi_n*Mg_i_n -K_DT*(1e-3*u_notBigg_ATP_mi_i_n)))
notBigg_J_MgADPi_n(u_notBigg_ADP_mi_i_n) = 1*mito_scale * (x_MgA*(ADP_fi_n*Mg_i_n -K_DD*(1e-3*u_notBigg_ADP_mi_i_n)))

Table J. Julia Fluxes (Part 2)

Fluxes
PDHm_n(u_pyr_m_n,u_coa_m_n,u_nad_m_n) = 1*(mito_scale *VmaxPDHCmito_n*(u_pyr_m_n<br>/(u_pyr_m_n+KmPyrMitoPDH_n))*(u_nad_m_n/(u_nad_m_n+KmNADmitoPDH_na))*(u_coa_m_n<br>/(u_coa_m_n+KmCoAmitoPDH_n)))
CSm_n(u_accoa_m_n,u_oaa_m_n,u_cit_m_n,u_coa_m_n) = 1*(mito_scale * VmaxCSmito_n<br>*(u_oaa_m_n/(u_oaa_m_n+KmOxaMito_n*(1.0+u_cit_m_n/KiCitMito_n)))*(u_accoa_m_n<br>/(u_accoa_m_n+KmAcCoAmito_n*(1.0+u_coa_m_n/KiCoA_n))))
ACONTm_n(u_icit_m_n,u_cit_m_n) = 1*(mito_scale * VmaxAco_n*(u_cit_m_n-u_icit_m_n<br>/KeqAco_na)/(1.0+u_cit_m_n/KmCitAco_n+u_icit_m_n/KmIsoCitAco_n))
ICDHxm_n(u_nadh_m_n,u_icit_m_n,u_nad_m_n) = 1*(mito_scale * VmaxIDH_n*(u_nad_m_n<br>/KiNADmito_na)*((u_icit_m_n/KmIsoCitIDHm_n)ñIDH)/(1.0+u_nad_m_n/KiNADmito_na<br>+(KmNADmito_na/KiNADmito_na)*((u_icit_m_n/KmIsoCitIDHm_n)ñIDH)+u_nadh_m_n/KiNADHmito_na<br>+(u_nad_m_n/KiNADmito_na)*((u_icit_m_n/KmIsoCitIDHm_n)ñIDH)+((KmNADmito_na*u_nadh_m_n<br>)/(KiNADmito_na*KiNADHmito_na))*((u_icit_m_n/KmIsoCitIDHm_n)ñIDH)))
AKGDm_n(u_coa_m_n,u_nadh_m_n,u_ca2_m_n,u_adp_m_n,u_succoa_m_n,u_nad_m_n,u_atp_m<br>_n,u_akg_m_n) = 1*((mito_scale * VmaxKGDH_n*(1+u_adp_m_n/KiADPmito_KGDH_n)*(u_akg_m_n<br>/Km1KGDHKGDH_n)*(u_coa_m_n/Km_CoA_kgdhKGDH_n)*(u_nad_m_n/KmNADkgdhKGDH_na))<br>/(((u_coa_m_n/Km_CoA_kgdhKGDH_n)*(u_nad_m_n/KmNADkgdhKGDH_na)*(u_akg_m_n<br>/Km1KGDHKGDH_n+(1+u_atp_m_n/KiATPmito_KGDH_n)/(1+u_ca2_m_n/KiCa2KGDH_n)))+((u_akg_m_n<br>/Km1KGDHKGDH_n)*(u_coa_m_n/Km_CoA_kgdhKGDH_n+u_nad_m_n/KmNADkgdhKGDH_na)*(1<br>+u_nadh_m_n/KiNADHKGDHKGDH_na+u_succoa_m_n/Ki_SucCoA_kgdhKGDH_n))))
SUCOASm_n(u_coa_m_n,u_succ_m_n,u_pi_m_n,u_adp_m_n,u_succoa_m_n,u_atp_m_n) = 1<br>*(mito_scale * VmaxSuccoaATPscs_n*(1+AmaxPscs_n*((u_pi_m_nñpscs_n)/((u_pi_m_nñpscs_n<br>)+(Km_pi_scs_nañpscs_n))))*(u_succoa_m_n*u_adp_m_n*u_pi_m_n-u_succ_m_n<br>*u_coa_m_n*u_atp_m_n/Keqsuccoascs_na)/((1+u_succoa_m_n/Km_succoa_scs_n)*(1<br>+u_adp_m_n/Km_ADPmito_scs_n)*(1+u_pi_m_n/Km_pi_scs_na)+(1+u_succ_m_n<br>/Km_succ_scs_n)*(1+u_coa_m_n/Km_coa_scs_n)*(1+u_atp_m_n/Km_atpmito_scs_n)))
FUMm_n(u_fum_m_n,u_mal_L_m_n) = 1*(mito_scale * Vmaxfum_n*(u_fum_m_n-u_mal_L_m_n<br>/Keqfummito_na)/(1.0+u_fum_m_n/Km_fummito_n+u_mal_L_m_n/Km_malmito_n))
MDHm_n(u_nadh_m_n,u_oaa_m_n,u_nad_m_n,u_mal_L_m_n) = 1*(mito_scale * VmaxMDHmito_n<br>*(u_mal_L_m_n*u_nad_m_n-u_oaa_m_n*u_nadh_m_n/Keqmdhmito_na)/((1.0+u_mal_L_m_n<br>/Km_mal_mdh_n)*(1.0+u_nad_m_n/Km_nad_mdh_na)+(1.0+u_oaa_m_n/Km_oxa_mdh_n)*(1.0<br>+u_nadh_m_n/Km_nadh_mdh_na)))
OCOAT1m_n(u_acac_c_n,u_aacoa_m_n,u_succoa_m_n,u_succ_m_n) = 1*mito_scale<br>* ((VmaxfSCOT_n*u_succoa_m_n*u_acac_c_n/(Ki_SucCoA_SCOT_n*Km_AcAc_SCOT_n<br>*(u_succoa_m_n/Ki_SucCoA_SCOT_n+Km_SucCoA_SCOT_n*u_acac_c_n/(Ki_SucCoA_SCOT_n<br>*Km_AcAc_SCOT_n)+u_aacoa_m_n/Ki_AcAcCoA_SCOT_n+Km_AcAcCoA_SCOT_n*u_succ_m_n<br>/(Ki_AcAcCoA_SCOT_n*Km_SUC_SCOT_n)+u_succoa_m_n*u_acac_c_n/(Ki_SucCoA_SCOT_n<br>*Km_AcAc_SCOT_n)+u_succoa_m_n*u_aacoa_m_n/(Ki_SucCoA_SCOT_n*Ki_AcAcCoA_SCOT_n)<br>+Km_SucCoA_SCOT_n*u_acac_c_n*u_succ_m_n/(Ki_SucCoA_SCOT_n*Km_AcAc_SCOT_n<br>*Ki_SUC_SCOT_n)+u_aacoa_m_n*u_succ_m_n/(Ki_AcAcCoA_SCOT_n*Km_SUC_SCOT_n))))<br>-(VmaxrSCOT_n*u_aacoa_m_n*u_succ_m_n/(Ki_AcAcCoA_SCOT_n*Km_SUC_SCOT_n*(u_succoa_m_n<br>/Ki_SucCoA_SCOT_n+Km_SucCoA_SCOT_n*u_acac_c_n/(Ki_SucCoA_SCOT_n*Km_AcAc_SCOT_n)<br>+u_aacoa_m_n/Ki_AcAcCoA_SCOT_n+Km_AcAcCoA_SCOT_n*u_succ_m_n/(Ki_AcAcCoA_SCOT_n<br>*Km_SUC_SCOT_n)+u_succoa_m_n*u_acac_c_n/(Ki_SucCoA_SCOT_n*Km_AcAc_SCOT_n)<br>+u_succoa_m_n*u_aacoa_m_n/(Ki_SucCoA_SCOT_n*Ki_AcAcCoA_SCOT_n)+Km_SucCoA_SCOT_n<br>*u_acac_c_n*u_succ_m_n/(Ki_SucCoA_SCOT_n*Km_AcAc_SCOT_n*Ki_SUC_SCOT_n)<br>+u_aacoa_m_n*u_succ_m_n/(Ki_AcAcCoA_SCOT_n*Km_SUC_SCOT_n)))))
ACACT1rm_n(u_aacoa_m_n,u_coa_m_n) = 1*mito_scale * (Vmax_thiolase_f_n*u_coa_m_n<br>*u_aacoa_m_n/(Ki_CoA_thiolase_f_n*Km_AcAcCoA_thiolase_f_n+Km_AcAcCoA_thiolase_f_n<br>*u_coa_m_n+Km_CoA_thiolase_f_n*u_aacoa_m_n+u_coa_m_n*u_aacoa_m_n))

Table K. Julia Fluxes (Part 3)

**Fluxes**
notBigg_JbHBTrArtCap(t,u_bhb_b_b) = (2*(C_bHB_a-u_bhb_b_b)/eto_b)*notBigg_FinDyn_W2017
notBigg_MCT1_bHB_b(u_bhb_e_e,u_bhb_b_b) = 1*(VmaxMCTbhb_b*(u_bhb_b_b/(u_bhb_b_b<br>+KmMCT1_bHB_b)-u_bhb_e_e/(u_bhb_e_e+KmMCT1_bHB_b)))
BHBt_n(u_bhb_c_n,u_bhb_e_e) = 1*(VmaxMCTbhb_n*(u_bhb_e_e/(u_bhb_e_e+KmMCT2_bHB_n)<br>-u_bhb_c_n/(u_bhb_c_n+KmMCT2_bHB_n)))
BHBt_a(u_bhb_c_a,u_bhb_e_e) = 1*(VmaxMCTbhb_a*(u_bhb_e_e/(u_bhb_e_e+KmMCT1_bHB_a)<br>-u_bhb_c_a/(u_bhb_c_a+KmMCT1_bHB_a)))
BDHm_n(u_acac_c_n,u_bhb_c_n,u_nadh_m_n,u_nad_m_n) = 1*mito_scale * (Vmax_bHBDH_f_n<br>*u_nad_m_n*u_bhb_c_n/(Ki_NAD_B_HBD_f_n*Km_betaHB_BHBD_n+Km_betaHB_BHBD_n<br>*u_nad_m_n+Km_NAD_B_HBD_n*u_bhb_c_n+u_nad_m_n*u_bhb_c_n)-(Vmax_bHBDH_r_n<br>*u_nadh_m_n*u_acac_c_n/(Ki_NADH_BHBD_r_n*Km_AcAc_BHBD_n+Km_AcAc_BHBD_n<br>*u_nadh_m_n+Km_NADH_BHBD_n*u_acac_c_n+u_nadh_m_n*u_acac_c_n)))
ASPTAm_n(u_oaa_m_n,u_akg_m_n,u_glu_L_m_n,u_asp_L_m_n) = 1*(mito_scale * VfAAT_n<br>*(u_asp_L_m_n*u_akg_m_n-u_oaa_m_n*u_glu_L_m_n/KeqAAT_n)/(KmAKG_AAT_n<br>*u_asp_L_m_n+KmASP_AAT_n*(1.0+u_akg_m_n/KiAKG_AAT_n)*u_akg_m_n+u_asp_L_m_n<br>*u_akg_m_n+KmASP_AAT_n*u_akg_m_n*u_glu_L_m_n/KiGLU_AAT_n+(KiASP_AAT_n*KmAKG_AAT_n<br>/(KmOXA_AAT_n*KiGLU_AAT_n))*(KmGLU_AAT_n*u_asp_L_m_n*u_oaa_m_n/KiASP_AAT_n<br>+u_oaa_m_n*u_glu_L_m_n+KmGLU_AAT_n*(1.0+u_akg_m_n/KiAKG_AAT_n)*u_oaa_m_n<br>+KmOXA_AAT_n*u_glu_L_m_n)))
MDH_n(u_nadh_c_n,u_mal_L_c_n,u_oaa_c_n,u_nad_c_n) = 1*(VmaxcMDH_n*(u_mal_L_c_n<br>*u_nad_c_n-u_oaa_c_n*u_nadh_c_n/Keqcmdh_n)/((1+u_mal_L_c_n/Kmmalcmdh_n)*(1<br>+u_nad_c_n/Kmnadcmdh_n)+(1+u_oaa_c_n/Kmoxacmdh_n)*(1+u_nadh_c_n/Kmnadhcmdh_n)-1))
ASPTA_n(u_glu_L_c_n,u_asp_L_c_n,u_oaa_c_n,u_akg_c_n) = 1*(VfCAAT_n*(u_asp_L_c_n<br>*u_akg_c_n-u_oaa_c_n*u_glu_L_c_n/KeqCAAT_n)/(KmAKG_CAAT_n*u_asp_L_c_n<br>+KmASP_CAAT_n*(1.0+u_akg_c_n/KiAKG_CAAT_n)*u_akg_c_n+u_asp_L_c_n*u_akg_c_n<br>+KmASP_CAAT_n*u_akg_c_n*u_glu_L_c_n/KiGLU_CAAT_n+(KiASP_CAAT_n*KmAKG_CAAT_n<br>/(KmOXA_CAAT_n*KiGLU_CAAT_n))*(KmGLU_CAAT_n*u_asp_L_c_n*u_oaa_c_n/KiASP_CAAT_n<br>+u_oaa_c_n*u_glu_L_c_n+KmGLU_CAAT_n*(1.0+u_akg_c_n/KiAKG_CAAT_n)*u_oaa_c_n<br>+KmOXA_CAAT_n*u_glu_L_c_n)))
ASPGLUm_n(u_h_c_n,u_glu_L_c_n,u_asp_L_m_n,u_asp_L_c_n,u_notBigg_MitoMembrPotent_m<br>_n,u_glu_L_m_n,u_h_m_n) = 1*mito_scale * (Vmaxagc_n*(u_asp_L_m_n*u_glu_L_c_n<br>-u_asp_L_c_n*u_glu_L_m_n/((exp(u_notBigg_MitoMembrPotent_m_n)^(F/(R*T)))*(u_h_c_n<br>/u_h_m_n)))/((u_asp_L_m_n+Km_aspmito_agc_n)*(u_glu_L_c_n+Km_glu_agc_n)<br>+(u_asp_L_c_n+Km_asp_agc_n)*(u_glu_L_m_n+Km_glumito_agc_n)))
AKGMALtm_n(u_mal_L_c_n,u_akg_m_n,u_akg_c_n,u_mal_L_m_n) = 1*mito_scale<br>* (Vmaxmakgc_n*(u_mal_L_c_n*u_akg_m_n-u_mal_L_m_n*u_akg_c_n)/((u_mal_L_c_n<br>+Km_mal_mkgc_n)*(u_akg_m_n+Km_akgmito_mkgc_n)+(u_mal_L_m_n+Km_malmito_mkgc_n)<br>*(u_akg_c_n+Km_akg_mkgc_n)))

Table L. Julia Fluxes (Part 4)

**Fluxes**
r0509_a(u_nadh_m_a,u_pi_m_a) = 1*mito_scale * (x_DH_a*(r_DH_a*NAD_x_a-(1e-3<br>*u_nadh_m_a))*((1+(1e-3*u_pi_m_a)/k_Pi1)/(1+(1e-3*u_pi_m_a)/k_Pi2)))
NADH2_u10mi_a(u_nadh_m_a,u_q10h2_m_a) = 1*mito_scale * (x_C1*(exp(-(dG_C1op_a+4<br>*dG_H_a)/etcRT)*(1e-3*u_nadh_m_a)*Q_a-NAD_x_a*(1e-3*u_q10h2_m_a)))
CYOR_u10mi_a(u_focytC_m_a,u_q10h2_m_a,u_pi_m_a,u_notBigg_MitoMembrPotent_m_a) = 1<br>*mito_scale * (x_C3*((1+(1e-3*u_pi_m_a)/k_Pi3)/(1+(1e-3*u_pi_m_a)/k_Pi4))*(exp(<br>-(dG_C3op_a+4*dG_H_a-2*etcF*u_notBigg_MitoMembrPotent_m_a)/(2*etcRT))*Cox_a*(1e-3<br>*u_q10h2_m_a)0̂.5-(1e-3*u_focytC_m_a)*Q_a0̂.5))
CYOOm2i_a(u_notBigg_Ctot_m_a,u_focytC_m_a,u_notBigg_MitoMembrPotent_m_a,u_o2_c_a<br>) = 1*mito_scale * (x_C4*((1e-3*u_o2_c_a)/((1e-3*u_o2_c_a)+k_O2))*((1e-3*u_focytC_m_a)<br>/(1e-3*u_notBigg_Ctot_m_a))*(exp(-(dG_C4op_a+2*dG_H_a)/(2*etcRT))*(1e-3*u_focytC_m_a)<br>*((1e-3*u_o2_c_a)0̂.25)-Cox_a*exp(etcF*u_notBigg_MitoMembrPotent_m_a/etcRT)))
ATPS4mi_a(u_notBigg_ATP_mx_m_a,u_pi_m_a,u_notBigg_ADP_mx_m_a) = 1*mito_scale<br>* (x_F1_a*(exp(-(dG_F1op_a-n_A*dG_H_a)/etcRT)*(K_DD_a/K_DT_a)*(1e-3<br>*u_notBigg_ADP_mx_m_a)*(1e-3*u_pi_m_a)-(1e-3*u_notBigg_ATP_mx_m_a)))
ATPtm_a(u_notBigg_MitoMembrPotent_m_a) = 1*mito_scale * (x_ANT_a*(ADP_fi_a/(ADP_fi_a<br>+ATP_fi_a*exp(-etcF*(0.35*u_notBigg_MitoMembrPotent_m_a)/etcRT))-ADP_fx_a/(ADP_fx_a<br>+ATP_fx_a*exp(-etcF*(-0.65*u_notBigg_MitoMembrPotent_m_a)/etcRT)))*(ADP_fi_a/(ADP_fi_a<br>+k_mADP_a)))
notBigg_J_Pi1_a(u_h_i_a,u_h_m_a) = 1*mito_scale * (x_Pi1*((1e-3*u_h_m_a)*H2PIi_a-(1e<br>-3*u_h_i_a)*H2PIx_a)/(H2PIi_a+k_PiH))
notBigg_J_Hle_a(u_h_i_a,u_h_m_a,u_notBigg_MitoMembrPotent_m_a) = 1*mito_scale<br>* (x_Hle*u_notBigg_MitoMembrPotent_m_a*((1e-3*u_h_i_a)*exp(etcF<br>*u_notBigg_MitoMembrPotent_m_a/etcRT)-(1e-3*u_h_m_a))/(exp(etcF<br>*u_notBigg_MitoMembrPotent_m_a/etcRT)-1))
notBigg_J_KH_a(u_h_i_a,u_h_m_a,u_k_m_a) = 1*mito_scale * (x_KH*(K_i*(1e-3*u_h_m_a<br>)-(1e-3*u_k_m_a)*(1e-3*u_h_i_a)))
notBigg_J_K_a(u_k_m_a,u_notBigg_MitoMembrPotent_m_a) = 1*mito_scale * (x_K<br>*u_notBigg_MitoMembrPotent_m_a*(K_i*exp(etcF*u_notBigg_MitoMembrPotent_m_a/etcRT)-(1e-3<br>*u_k_m_a))/(exp(etcF*u_notBigg_MitoMembrPotent_m_a/etcRT)-1))

Table M. Julia Fluxes (Part 5)

**Fluxes**
ADK1m_a(u_atp_i_a,u_adp_i_a,u_amp_i_a) = 1*mito_scale * (x_AK*(K_AK*(1e-3*u_adp_i_a<br>)*(1e-3*u_adp_i_a)-(1e-3*u_amp_i_a)*(1e-3*u_atp_i_a)))
notBigg_J_AMP_a(u_amp_i_a,u_amp_c_a) = 1*mito_scale * (gamma*x_A*((1e-3*u_amp_c_a)<br>-(1e-3*u_amp_i_a)))
notBigg_J_ADP_a(u_adp_c_a,u_adp_i_a) = 1*mito_scale * (gamma*x_A*((1e-3*u_adp_c_a)<br>-(1e-3*u_adp_i_a)))
notBigg_J_ATP_a(u_atp_i_a,u_atp_c_a) = 1*mito_scale * (gamma*x_A*((1e-3*u_atp_c_a)<br>-(1e-3*u_atp_i_a)))
notBigg_J_Pi2_a(u_pi_i_a,u_pi_c_a) = 1*mito_scale * (gamma*x_Pi2*(1e-3*u_pi_c_a-(1e-3<br>*u_pi_i_a)))
notBigg_J_Ht_a(u_h_i_a,u_h_c_a) = 1*mito_scale * (gamma*x_Ht*(1e-3*u_h_c_a-(1e-3<br>*u_h_i_a)))
notBigg_J_MgATPx_a(u_notBigg_ATP_mx_m_a,u_mg2_m_a) = 1*mito_scale * (x_MgA*(ATP_fx_a<br>*(1e-3*u_mg2_m_a)-K_DT_a*(1e-3*u_notBigg_ATP_mx_m_a)))
notBigg_J_MgADPx_a(u_notBigg_ADP_mx_m_a,u_mg2_m_a) = 1*mito_scale * (x_MgA*(ADP_fx_a<br>*(1e-3*u_mg2_m_a)-K_DD_a*(1e-3*u_notBigg_ADP_mx_m_a)))
notBigg_J_MgATPi_a(u_notBigg_ATP_mi_i_a) = 1*mito_scale * (x_MgA*(ATP_fi_a*Mg_i_a<br>-K_DT_a*(1e-3*u_notBigg_ATP_mi_i_a)))
notBigg_J_MgADPi_a(u_notBigg_ADP_mi_i_a) = 1*mito_scale * (x_MgA*(ADP_fi_a*Mg_i_a<br>-K_DD_a*(1e-3*u_notBigg_ADP_mi_i_a)))

Table N. Julia Fluxes (Part 6)

**Fluxes**
PDHm_a(u_nad_m_a,u_pyr_m_a,u_coa_m_a) = 1*mito_scale * (VmaxPDHCmito_a*(u_pyr_m_a<br>/(u_pyr_m_a+KmPyrMitoPDH_a))*(u_nad_m_a/(u_nad_m_a+KmNADmitoPDH_na))*(u_coa_m_a<br>/(u_coa_m_a+KmCoAmitoPDH_a)))
CSm_a(u_oaa_m_a,u_cit_m_a,u_accoa_m_a,u_coa_m_a) = 1*mito_scale * (VmaxCSmito_a<br>*(u_oaa_m_a/(u_oaa_m_a+KmOxaMito_a*(1.0+u_cit_m_a/KiCitMito_a)))*(u_accoa_m_a<br>/(u_accoa_m_a+KmAcCoAmito_a*(1.0+u_coa_m_a/KiCoA_a))))
ACONTm_a(u_cit_m_a,u_icit_m_a) = 1*mito_scale * (VmaxAco_a*(u_cit_m_a-u_icit_m_a<br>/KeqAco_na)/(1.0+u_cit_m_a/KmCitAco_a+u_icit_m_a/KmIsoCitAco_a))
ICDHxm_a(u_nad_m_a,u_nadh_m_a,u_icit_m_a) = 1*mito_scale * (VmaxIDH_a*(u_nad_m_a<br>/KiNADmito_na)*((u_icit_m_a/KmIsoCitIDHm_a)ñIDH)/(1.0+u_nad_m_a/KiNADmito_na<br>+(KmNADmito_na/KiNADmito_na)*((u_icit_m_a/KmIsoCitIDHm_a)ñIDH)+u_nadh_m_a/KiNADHmito_na<br>+(u_nad_m_a/KiNADmito_na)*((u_icit_m_a/KmIsoCitIDHm_a)ñIDH)+((KmNADmito_na*u_nadh_m_a<br>)/(KiNADmito_na*KiNADHmito_na))*((u_icit_m_a/KmIsoCitIDHm_a)ñIDH)))
AKGDm_a(u_coa_m_a,u_nadh_m_a,u_akg_m_a,u_ca2_m_a,u_nad_m_a,u_succoa_m_a,u_atp_m<br>_a,u_adp_m_a) = 1*mito_scale * ((VmaxKGDH_a*(1+u_adp_m_a/KiADPmito_KGDH_a)*(u_akg_m_a<br>/Km1KGDHKGDH_a)*(u_coa_m_a/Km_CoA_kgdhKGDH_a)*(u_nad_m_a/KmNADkgdhKGDH_na))<br>/(((u_coa_m_a/Km_CoA_kgdhKGDH_a)*(u_nad_m_a/KmNADkgdhKGDH_na)*(u_akg_m_a<br>/Km1KGDHKGDH_a+(1+u_atp_m_a/KiATPmito_KGDH_a)/(1+u_ca2_m_a/KiCa2KGDH_a)))+((u_akg_m_a<br>/Km1KGDHKGDH_a)*(u_coa_m_a/Km_CoA_kgdhKGDH_a+u_nad_m_a/KmNADkgdhKGDH_na)*(1<br>+u_nadh_m_a/KiNADHKGDHKGDH_na+u_succoa_m_a/Ki_SucCoA_kgdhKGDH_a))))
SUCOASm_a(u_coa_m_a,u_succoa_m_a,u_atp_m_a,u_succ_m_a,u_pi_m_a,u_adp_m_a) = 1<br>*mito_scale * (VmaxSuccoaATPscs_a*(1+AmaxPscs_a*((u_pi_m_añpscs_a)/((u_pi_m_añpscs_a<br>)+(Km_pi_scs_nañpscs_a))))*(u_succoa_m_a*u_adp_m_a*u_pi_m_a-u_succ_m_a<br>*u_coa_m_a*u_atp_m_a/Keqsuccoascs_na)/((1+u_succoa_m_a/Km_succoa_scs_a)*(1<br>+u_adp_m_a/Km_ADPmito_scs_a)*(1+u_pi_m_a/Km_pi_scs_na)+(1+u_succ_m_a<br>/Km_succ_scs_a)*(1+u_coa_m_a/Km_coa_scs_a)*(1+u_atp_m_a/Km_atpmito_scs_a)))
FUMm_a(u_mal_L_m_a,u_fum_m_a) = 1*mito_scale * (Vmaxfum_a*(u_fum_m_a-u_mal_L_m_a<br>/Keqfummito_na)/(1.0+u_fum_m_a/Km_fummito_a+u_mal_L_m_a/Km_malmito_a))
MDHm_a(u_nad_m_a,u_oaa_m_a,u_mal_L_m_a,u_nadh_m_a) = 1*mito_scale * (VmaxMDHmito_a<br>*(u_mal_L_m_a*u_nad_m_a-u_oaa_m_a*u_nadh_m_a/Keqmdhmito_na)/((1.0+u_mal_L_m_a<br>/Km_mal_mdh_a)*(1.0+u_nad_m_a/Km_nad_mdh_na)+(1.0+u_oaa_m_a/Km_oxa_mdh_a)*(1.0<br>+u_nadh_m_a/Km_nadh_mdh_na)))
PCm_a(u_co2_m_a,u_oaa_m_a,u_pyr_m_a,u_atp_m_a,u_adp_m_a) = 1*mito_scale<br>* (((u_atp_m_a/u_adp_m_a)/(muPYRCARB_a+(u_atp_m_a/u_adp_m_a)))*VmPYRCARB_a<br>*(u_pyr_m_a*u_co2_m_a-u_oaa_m_a/KeqPYRCARB_a)/(KmPYR_PYRCARB_a*KmCO2_PYRCARB_a<br>+KmPYR_PYRCARB_a*u_co2_m_a+KmCO2_PYRCARB_a*u_pyr_m_a+u_co2_m_a*u_pyr_m_a))
GLNt4_n(u_gln_L_c_n,u_gln_L_e_e) = 1*(TmaxSNAT_GLN_n*(u_gln_L_e_e-u_gln_L_c_n<br>/coeff_gln_ratio_n_ecs)/(KmSNAT_GLN_n+u_gln_L_c_n))

Table O. Julia Fluxes (Part 7)

**Fluxes**
GLUNm_n(u_gln_L_c_n,u_glu_L_m_n) = 1*mito_scale * (mito_scale * VmGLS_n*(u_gln_L_c_n-u_glu_L_m_n/KeqGLS_n)/(KmGLNGLS_n*(1.0+u_glu_L_m_n/KiGLUGLS_n)+u_gln_L_c_n))
GLUt6_a(u_na1_c_a,u_notBigg_Va_c_a,u_k_e_e,u_glu_L_syn_syn,u_k_c_a,u_glu_L_c_a) = 1*(-((1/(2*F*1e-3))*(-alpha_EAAT*exp(-beta_EAAT*(u_notBigg_Va_c_a-((R*T/(2*F*1e-3))*log(((Na_syn_EAAT/u_na1_c_a)^3)*(H_syn_EAAT/H_ast_EAAT)*(u_glu_L_syn_syn/u_glu_L_c_a)*(u_k_c_a/u_k_e_e))))))))
GLUDxm_a(u_nad_m_a,u_nadh_m_a,u_glu_L_c_a,u_akg_m_a) = 1*mito_scale * (VmGDH_a*(u_nad_m_a*u_glu_L_c_a-u_nadh_m_a*u_akg_m_a/KeqGDH_a)/(KiNAD_GDH_a*KmGLU_GDH_a+KmGLU_GDH_a*u_nad_m_a+KiNAD_GDH_a*u_glu_L_c_a+u_glu_L_c_a*u_nad_m_a+u_glu_L_c_a*u_nad_m_a/KiAKG_GDH_a+KiNAD_GDH_a*KmGLU_GDH_a*u_nadh_m_a/KiNADH_GDH_a+KiNAD_GDH_a*KmGLU_GDH_a*KmNADH_GDH_a*u_akg_m_a/(KiAKG_GDH_a*KiNADH_GDH_a)+KmNADH_GDH_a*u_glu_L_c_a*u_nadh_m_a/KiNADH_GDH_a+KiNAD_GDH_a*KmGLU_GDH_a*KmAKG_GDH_a*u_nadh_m_a/(KiAKG_GDH_a*KiNADH_GDH_a)+KiNAD_GDH_a*KmGLU_GDH_a*KmAKG_GDH_a*u_akg_m_a*u_nadh_m_a/(KiAKG_GDH_a*KiNADH_GDH_a)+u_glu_L_c_a*u_nad_m_a*u_akg_m_a/KiAKG_GDH_a+KiNAD_GDH_a*KmGLU_GDH_a*KmAKG_GDH_a/KiAKG_GDH_a+KiNAD_GDH_a*KmGLU_GDH_a*u_glu_L_c_a*u_nadh_m_a*u_akg_m_a/(KiGLU_GDH_a*KiAKG_GDH_a*KiNADH_GDH_a)+KiNAD_GDH_a*KmGLU_GDH_a*u_akg_m_a*u_nadh_m_a/(KiAKG_GDH_a*KiNADH_GDH_a)+KmNADH_GDH_a*KmGLU_GDH_a*u_akg_m_a*u_nad_m_a/(KiAKG_GDH_a*KiNADH_GDH_a)))
GLNS_a(u_adp_c_a,u_atp_c_a,u_glu_L_c_a) = 1*(VmaxGLNsynth_a*(u_glu_L_c_a/(KmGLNsynth_a+u_glu_L_c_a))*((1/(u_atp_c_a/u_adp_c_a))/(muGLNsynth_a+(1/(u_atp_c_a/u_adp_c_a)))))
GLNt4_a(u_gln_L_c_a,u_gln_L_e_e) = 1*(TmaxSNAT_GLN_a*(u_gln_L_c_a-u_gln_L_e_e)/(KmSNAT_GLN_a+u_gln_L_c_a))
notBigg_JdHbin(t,u_o2_b_b) = 2*(C_0_a-u_o2_b_b)*notBigg_FinDyn_W2017
notBigg_JdHbout(t,u_notBigg_ddHb_b_b) = (u_notBigg_ddHb_b_b/u_notBigg_vV_b_b)*notBigg_Fout_W2017
notBigg_JO2art2cap(t,u_o2_b_b) = (1/eto_b)*2*(C_0_a-u_o2_b_b)*notBigg_FinDyn_W2017
notBigg_JO2fromCap2a(u_o2_b_b,u_o2_c_a) = 1*((PScapA*(KO2b*(HbOP/u_o2_b_b-1.)^(-1/param_degree_nh)-u_o2_c_a)))
notBigg_JO2fromCap2n(u_o2_b_b,u_o2_c_n) = 1*((PScapNAratio*PScapA*(KO2b*(HbOP/u_o2_b_b-1.)^(-1/param_degree_nh)-u_o2_c_n)))

Table P. Julia Fluxes (Part 8)

**Fluxes**
notBigg_trGLC_art_cap(t,u_glc_D_b_b) = (1/eto_b)*(2*(C_Glc_a-u_glc_D_b_b))<br>*notBigg_FinDyn_W2017
notBigg_JGlc_be(u_glc_D_ecsEndothelium_ecsEndothelium,u_glc_D_b_b) = 1*(TmaxGLCce<br>*(u_glc_D_b_b*(KeG+u_glc_D_ecsEndothelium_ecsEndothelium)<br>-u_glc_D_ecsEndothelium_ecsEndothelium*(KeG+u_glc_D_b_b))/(KeG^2+KeG*ReGoi*u_glc_D_b_b<br>+KeG*ReGio*u_glc_D_ecsEndothelium_ecsEndothelium+ReGee*u_glc_D_b_b<br>*u_glc_D_ecsEndothelium_ecsEndothelium))
notBigg_JGlc_e2ecsBA(u_glc_D_ecsBA_ecsBA,u_glc_D_ecsEndothelium_ecsEndothelium) = 1<br>*(TmaxGLCeb*(u_glc_D_ecsEndothelium_ecsEndothelium*(KeG2+u_glc_D_ecsBA_ecsBA)<br>-u_glc_D_ecsBA_ecsBA*(KeG2+u_glc_D_ecsEndothelium_ecsEndothelium))/(KeG2^2+KeG2*ReGoi2<br>*u_glc_D_ecsEndothelium_ecsEndothelium+KeG2*ReGio2*u_glc_D_ecsBA_ecsBA+ReGee2<br>*u_glc_D_ecsEndothelium_ecsEndothelium*u_glc_D_ecsBA_ecsBA))
notBigg_JGlc_ecsBA2a(u_glc_D_ecsBA_ecsBA,u_glc_D_c_a) = 1*(TmaxGLCba<br>*(u_glc_D_ecsBA_ecsBA*(KeG3+u_glc_D_c_a)-u_glc_D_c_a*(KeG3+u_glc_D_ecsBA_ecsBA))<br>/(KeG3^2+KeG3*ReGoi3*u_glc_D_ecsBA_ecsBA+KeG3*ReGio3*u_glc_D_c_a+ReGee3<br>*u_glc_D_ecsBA_ecsBA*u_glc_D_c_a))
notBigg_JGlc_a2ecsAN(u_glc_D_ecsAN_ecsAN,u_glc_D_c_a) = 1*(TmaxGLCai*(u_glc_D_c_a<br>*(KeG4+u_glc_D_ecsAN_ecsAN)-u_glc_D_ecsAN_ecsAN*(KeG4+u_glc_D_c_a))/(KeG4^2+KeG4<br>*ReGoi4*u_glc_D_c_a+KeG4*ReGio4*u_glc_D_ecsAN_ecsAN+ReGee4*u_glc_D_c_a<br>*u_glc_D_ecsAN_ecsAN))
notBigg_JGlc_ecsAN2n(u_glc_D_c_n,u_glc_D_ecsAN_ecsAN) = 1*(TmaxGLCin<br>*(u_glc_D_ecsAN_ecsAN*(KeG5+u_glc_D_c_n)-u_glc_D_c_n*(KeG5+u_glc_D_ecsAN_ecsAN))<br>/(KeG5^2+KeG5*ReGoi5*u_glc_D_ecsAN_ecsAN+KeG5*ReGio5*u_glc_D_c_n+ReGee5<br>*u_glc_D_ecsAN_ecsAN*u_glc_D_c_n))
notBigg_JGlc_diffEcs(u_glc_D_ecsBA_ecsBA,u_glc_D_ecsAN_ecsAN) = 1*(kGLCdiff<br>*(u_glc_D_ecsBA_ecsBA-u_glc_D_ecsAN_ecsAN))
GLCS2_a(u_notBigg_GS_c_a,u_udpg_c_a) = 1*(kL2_GS_a*u_notBigg_GS_c_a*u_udpg_c_a<br>/(kmL2_GS_a+u_udpg_c_a))
GLCP_a(u_glycogen_c_a,u_notBigg_GPa_c_a,u_camp_c_a,u_notBigg_GPb_c_a) = 1<br>*((u_notBigg_GPa_c_a/(u_notBigg_GPa_c_a+u_notBigg_GPb_c_a))*VmaxGP_a*u_glycogen_c_a<br>*(1/(1+(KmGP_AMP_a^nGPa)/(u_camp_c_a^nGPa))))
PGMT_a(u_g1p_c_a,u_g6p_c_a) = 1*((Vmaxfpglm_a*u_g1p_c_a/KmG1PPGLM_a-((Vmaxfpglm_a<br>*KmG6PPGLM_a)/(KmG1PPGLM_a*KeqPGLM_a))*u_g6p_c_a/KmG6PPGLM_a)/(1.0+u_g1p_c_a/KmG1PPGLM_a<br>+u_g6p_c_a/KmG6PPGLM_a))

Table Q. Julia Fluxes (Part 9)

**Fluxes**
PDE1_a(u_camp_c_a) = 1*(VmaxPDE_a*u_camp_c_a/(Kmcamppde_a+u_camp_c_a))
GALUi_a(u_ppi_c_a,u_g1p_c_a,u_udpg_c_a,u_utp_c_a) = 1*((VmaxfUDPGP*u_utp_c_a<br>*u_g1p_c_a/(KutpUDPGP*Kg1pUDPGP)-VmaxrUDPGP*u_ppi_c_a*u_udpg_c_a/(KpiUDPGP<br>*KUDPglucoUDPGP_a))/(1.0+u_g1p_c_a/Kg1pUDPGP+u_utp_c_a/KutpUDPGP+(u_g1p_c_a*u_utp_c_a)<br>/(Kg1pUDPGP*KutpUDPGP)+u_udpg_c_a/KUDPglucoUDPGP_a+u_ppi_c_a/KpiUDPGP+(u_ppi_c_a<br>*u_udpg_c_a)/(KpiUDPGP*KUDPglucoUDPGP_a)))
notBigg_psiGSAJay_a(u_notBigg_GS_c_a,u_udpg_c_a,u_notBigg_PHKa_c_a,u_notBigg_PKAa_c_a)<br>= 1*(((kg8_GSAJay*PP1_a0*(st_GSAJay-u_notBigg_GS_c_a))/((kmg8_GSAJay/(1.0+s1_GSAJay<br>*u_udpg_c_a/kg2_GSAJay))+(st_GSAJay-u_notBigg_GS_c_a)))-((kg7_GSAJay<br>*(u_notBigg_PHKa_c_a+u_notBigg_PKAa_c_a)*u_notBigg_GS_c_a)/(kmg7_GSAJay*(1+s1_GSAJay<br>*u_udpg_c_a/kg2_GSAJay)+u_notBigg_GS_c_a)))
notBigg_psiPHK_a(u_ca2_c_a,u_glycogen_c_a,u_g1p_c_a,u_notBigg_PHKa_c_a,u_notBigg_GP<br>a_c_a,u_udpg_c_a) = 1*((u_ca2_c_a/cai0_ca_ion)*(((kg5_PHK*u_notBigg_PHKa_c_a<br>*(pt_PHK-u_notBigg_GPa_c_a))/(kmg5_PHK*(1.0+s1_PHK*u_g1p_c_a/kg2_PHK)+(pt_PHK<br>-u_notBigg_GPa_c_a)))-((kg6_PHK*PP1_a0*u_notBigg_GPa_c_a)/(kmg6_PHK/(1+s2_PHK<br>*u_udpg_c_a/kgi_PHK)+u_notBigg_GPa_c_a))-((0.003198/(1+u_glycogen_c_a)+kmind_PHK)<br>*PP1_a0*u_notBigg_GPa_c_a)))
HEX1_n(u_glc_D_c_n,u_atp_c_n,u_g6p_c_n) = 1*((VmaxHK_n*u_glc_D_c_n/(u_glc_D_c_n<br>+KmHK_n))*(u_atp_c_n/(1+(u_atp_c_n/KIATPhex_n)ñHhexn))*(1/(1+u_g6p_c_n/KiHKG6P_n)))
HEX1_a(u_atp_c_a,u_g6p_c_a,u_glc_D_c_a) = 1*((VmaxHK_a*u_glc_D_c_a/(u_glc_D_c_a<br>+KmHK_a))*(u_atp_c_a/(1+(u_atp_c_a/KIATPhex_a)ñHhexa))*(1/(1+u_g6p_c_a/KiHKG6P_a)))
PGI_n(u_f6p_c_n,u_g6p_c_n) = 1*((Vmax_fPGI_n*(u_g6p_c_n/Km_G6P_fPGI_n-0.9<br>*u_f6p_c_n/Km_F6P_rPGI_n))/(1.0+u_g6p_c_n/Km_G6P_fPGI_n+u_f6p_c_n/Km_F6P_rPGI_n))
PGI_a(u_g6p_c_a,u_f6p_c_a) = 1*((Vmax_fPGI_a*(u_g6p_c_a/Km_G6P_fPGI_a-0.9<br>*u_f6p_c_a/Km_F6P_rPGI_a))/(1.0+u_g6p_c_a/Km_G6P_fPGI_a+u_f6p_c_a/Km_F6P_rPGI_a))
PFK_n(u_f6p_c_n,u_atp_c_n) = 1*(VmaxPFK_n*(u_atp_c_n/(1+(u_atp_c_n/KiPFK_ATP_na<br>)ñPFKn))*(u_f6p_c_n/(u_f6p_c_n+KmPFKF6P_n)))
PFK_a(u_atp_c_a,u_f26bp_c_a,u_f6p_c_a) = 1*(VmaxPFK_a*(u_atp_c_a/(1+(u_atp_c_a<br>/KiPFK_ATP_a)ñPFKa))*(u_f6p_c_a/(u_f6p_c_a+KmPFKF6P_a*(1-KoPFK_f26bp_a<br>*((u_f26bp_c_añPFKf26bp_a)/(KmF26BP_PFK_añPFKf26bp_a+u_f26bp_c_añPFKf26bp_a)))))<br>*(u_f26bp_c_a/(KmF26BP_PFK_a+u_f26bp_c_a)))

Table R. Julia Fluxes (Part 10)

Fluxes
PFK26_a(u_adp_c_a,u_atp_c_a,u_f26bp_c_a,u_f6p_c_a) = 1*(Vmax_PFKII_g*u_f6p_c_a *u_atp_c_a*u_adp_c_a/((u_f6p_c_a+Kmf6pPFKII_g)*(u_atp_c_a+KmatpPFKII_g)*(u_adp_c_a +Km_act_adpPFKII_g))-(Vmax_PFKII_g*u_f26bp_c_a/(u_f26bp_c_a+Km_f26bp_f_26pase_g*(1 +u_f6p_c_a/Ki_f6p_f_26_pase_g))))
FBA_n(u_fdp_c_n,u_dhap_c_n,u_g3p_c_n) = 1*(Vmaxald_n*(u_fdp_c_n-u_g3p_c_n *u_dhap_c_n/Keqald_n)/((1+u_fdp_c_n/KmfbpAld_n)+(1+u_g3p_c_n/KmgapAld_n)*(1 +u_dhap_c_n/KmdhapAld_n)-1))
FBA_a(u_g3p_c_a,u_fdp_c_a,u_dhap_c_a) = 1*(Vmaxald_a*(u_fdp_c_a-u_g3p_c_a *u_dhap_c_a/Keqald_a)/((1+u_fdp_c_a/KmfbpAld_a)+(1+u_g3p_c_a/KmgapAld_a)*(1 +u_dhap_c_a/KmdhapAld_a)-1))
TPI_n(u_dhap_c_n,u_g3p_c_n) = 1*(Vmaxtpi_n*(u_dhap_c_n-u_g3p_c_n/Keqtpi_n)/(1 +u_dhap_c_n/KmdhapTPI_n+u_g3p_c_n/KmgapTPI_n))
TPI_a(u_g3p_c_a,u_dhap_c_a) = 1*(Vmaxtpi_a*(u_dhap_c_a-u_g3p_c_a/Keqtpi_a)/(1 +u_dhap_c_a/KmdhapTPI_a+u_g3p_c_a/KmgapTPI_a))
GAPD_n(u_nadh_c_n,u_nad_c_n,u_g3p_c_n,u_pi_c_n,u_13dpg_c_n) = 1*(Vmaxgapdh_n *(u_nad_c_n*u_g3p_c_n*u_pi_c_n-u_13dpg_c_n*u_nadh_c_n/Keqgapdh_na)/((1+u_nad_c_n /KmnadGpdh_n)*(1+u_g3p_c_n/KmGapGapdh_n)*(1+u_pi_c_n/KmpiGpdh_n)+(1+u_nadh_c_n /KmnadhGapdh_n)*(1+u_13dpg_c_n/KmBPG13Gapdh_n)-1))
GAPD_a(u_nadh_c_a,u_nad_c_a,u_pi_c_a,u_13dpg_c_a,u_g3p_c_a) = 1*(Vmaxgapdh_a *(u_nad_c_a*u_g3p_c_a*u_pi_c_a-u_13dpg_c_a*u_nadh_c_a/Keqgapdh_na)/((1+u_nad_c_a /KmnadGpdh_a)*(1+u_g3p_c_a/KmGapGapdh_a)*(1+u_pi_c_a/KmpiGpdh_a)+(1+u_nadh_c_a /KmnadhGapdh_a)*(1+u_13dpg_c_a/KmBPG13Gapdh_a)-1))
PGK_n(u_13dpg_c_n,u_3pg_c_n,u_atp_c_n,u_adp_c_n) = 1*(Vmaxpgk_n*(u_13dpg_c_n *u_adp_c_n-u_3pg_c_n*u_atp_c_n/Keqpgk_na)/((1+u_13dpg_c_n/Kmbpg13pgk_n)*(1 +u_adp_c_n/Kmadppgk_n)+(1+u_3pg_c_n/Kmpg3pgk_n)*(1+u_atp_c_n/Kmatppgk_n)-1))
PGK_a(u_adp_c_a,u_atp_c_a,u_3pg_c_a,u_13dpg_c_a) = 1*(Vmaxpgk_a*(u_13dpg_c_a *u_adp_c_a-u_3pg_c_a*u_atp_c_a/Keqpgk_na)/((1+u_13dpg_c_a/Kmbpg13pgk_a)*(1 +u_adp_c_a/Kmadppgk_a)+(1+u_3pg_c_a/Kmpg3pgk_a)*(1+u_atp_c_a/Kmatppgk_a)-1))
PGM_n(u_2pg_c_n,u_3pg_c_n) = 1*(Vmaxpgm_n*(u_3pg_c_n-u_2pg_c_n/Keqpgm_n)/((1 +u_3pg_c_n/Kmpg3pgm_n)+(1+u_2pg_c_n/Kmpg2pgm_n)-1))

Table S. Julia Fluxes (Part 11)

Fluxes
PGM_a(u_2pg_c_a,u_3pg_c_a) = 1*(Vmaxpgm_a*(u_3pg_c_a-u_2pg_c_a/Keqpgm_a)/((1 +u_3pg_c_a/Kmpg3pgm_a)+(1+u_2pg_c_a/Kmpg2pgm_a)-1))
ENO_n(u_pep_c_n,u_2pg_c_n) = 1*(Vmaxenol_n*(u_2pg_c_n-u_pep_c_n/Keqenol_n)/((1 +u_2pg_c_n/Kmpg2enol_n)+(1+u_pep_c_n/Km_pep_enol_n)-1))
ENO_a(u_2pg_c_a,u_pep_c_a) = 1*(Vmaxenol_a*(u_2pg_c_a-u_pep_c_a/Keqenol_a)/((1 +u_2pg_c_a/Kmpg2enol_a)+(1+u_pep_c_a/Km_pep_enol_a)-1))
PYK_n(u_pep_c_n,u_atp_c_n,u_adp_c_n) = 1*(Vmaxpk_n*u_pep_c_n*u_adp_c_n /((u_pep_c_n+Km_pep_pk_n)*(u_adp_c_n+Km_adp_pk_n*(1+u_atp_c_n/Ki_ATP_pk_n))))
PYK_a(u_adp_c_a,u_atp_c_a,u_pep_c_a) = 1*(Vmaxpk_a*u_pep_c_a*u_adp_c_a /((u_pep_c_a+Km_pep_pk_a)*(u_adp_c_a+Km_adp_pk_a*(1+u_atp_c_a/Ki_ATP_pk_a))))
notBigg_JLacTr_b(u_lac_L_b_b,t) = (2*(C_Lac_a-u_lac_L_b_b)/eto_b) *notBigg_FinDyn_W2017
notBigg_MCT1_LAC_b(u_lac_L_b_b,u_lac_L_e_e) = 1*(TbLac*(u_lac_L_b_b/(u_lac_L_b_b +KbLac)-u_lac_L_e_e/(u_lac_L_e_e+KbLac)))
L_LACt2r_a(u_lac_L_e_e,u_lac_L_c_a) = 1*(TaLac*(u_lac_L_e_e/(u_lac_L_e_e +Km_Lac_a)-u_lac_L_c_a/(u_lac_L_c_a+Km_Lac_a)))
L_LACt2r_n(u_lac_L_e_e,u_lac_L_c_n) = 1*(TnLac*(u_lac_L_e_e/(u_lac_L_e_e +Km_LacTr_n)-u_lac_L_c_n/(u_lac_L_c_n+Km_LacTr_n)))
notBigg_jLacDiff_e(u_lac_L_e_e) = 0 1*(betaLacDiff*(u0_ss[150]-u_lac_L_e_e))

Table T. Julia Fluxes (Part 12)

**Fluxes**
notBigg_vLACgc(u_lac_L_b_b,u_lac_L_c_a) = 1*(TMaxLACgc*(u_lac_L_b_b/(u_lac_L_b_b +KtLACgc)-u_lac_L_c_a/(u_lac_L_c_a+KtLACgc)))
LDH_L_a(u_nad_c_a,u_nadh_c_a,u_lac_L_c_a,u_pyr_c_a) = 1*(VmfLDH_a*u_pyr_c_a *u_nadh_c_a-KeLDH_a*VmfLDH_a*u_lac_L_c_a*u_nad_c_a)
LDH_L_n(u_nad_c_n,u_lac_L_c_n,u_nadh_c_n,u_pyr_c_n) = 1*(VmfLDH_n*u_pyr_c_n *u_nadh_c_n-KeLDH_n*VmfLDH_n*u_lac_L_c_n*u_nad_c_n)
G6PDH2r_n(u_g6p_c_n,u_nadph_c_n,u_nadp_c_n,u_6pgl_c_n) = 1*(VmaxG6PDH_n*(1 /(K_G6P_G6PDH_n*K_NADP_G6PDH_n))*((u_g6p_c_n*u_nadp_c_n-u_6pgl_c_n*u_nadph_c_n /KeqG6PDH_n)/((1+u_g6p_c_n/K_G6P_G6PDH_n)*(1+u_nadp_c_n/K_NADP_G6PDH_n)+(1 +u_6pgl_c_n/K_GL6P_G6PDH_n)*(1+u_nadph_c_n/K_NADPH_G6PDH_n)-1)))
G6PDH2r_a(u_6pgl_c_a,u_nadp_c_a,u_g6p_c_a,u_nadph_c_a) = 1*(VmaxG6PDH_a*(1 /(K_G6P_G6PDH_a*K_NADP_G6PDH_a))*((u_g6p_c_a*u_nadp_c_a-u_6pgl_c_a*u_nadph_c_a /KeqG6PDH_a)/((1+u_g6p_c_a/K_G6P_G6PDH_a)*(1+u_nadp_c_a/K_NADP_G6PDH_a)+(1 +u_6pgl_c_a/K_GL6P_G6PDH_a)*(1+u_nadph_c_a/K_NADPH_G6PDH_a)-1)))
PGL_n(u_6pgc_c_n,u_6pgl_c_n) = 1*(Vmax6PGL_n*(1/K_GL6P_6PGL_n)*((u_6pgl_c_n -u_6pgc_c_n/Keq6PGL_n)/((1+u_6pgl_c_n/K_GL6P_6PGL_n)+(1+u_6pgc_c_n/K_GO6P_6PGL_n)-1)))
PGL_a(u_6pgc_c_a,u_6pgl_c_a) = 1*(Vmax6PGL_a*(1/K_GL6P_6PGL_a)*((u_6pgl_c_a -u_6pgc_c_a/Keq6PGL_a)/((1+u_6pgl_c_a/K_GL6P_6PGL_a)+(1+u_6pgc_c_a/K_GO6P_6PGL_a)-1)))
GND_n(u_6pgc_c_n,u_nadph_c_n,u_nadp_c_n,u_ru5p_D_c_n) = 1*(Vmax6PGDH_n*(1 /(K_GO6P_6PGDH_n*K_NADP_6PGDH_n))*(u_6pgc_c_n*u_nadp_c_n-u_ru5p_D_c_n *u_nadph_c_n/Keq6PGDH_n)/((1+u_6pgc_c_n/K_GO6P_6PGDH_n)*(1+u_nadp_c_n /K_NADP_6PGDH_n)+(1+u_ru5p_D_c_n/K_RU5P_6PGDH_n)*(1+u_nadph_c_n/K_NADPH_6PGDH_n)-1))
GND_a(u_6pgc_c_a,u_nadp_c_a,u_ru5p_D_c_a,u_nadph_c_a) = 1*(Vmax6PGDH_a*(1 /(K_GO6P_6PGDH_a*K_NADP_6PGDH_a))*(u_6pgc_c_a*u_nadp_c_a-u_ru5p_D_c_a *u_nadph_c_a/Keq6PGDH_a)/((1+u_6pgc_c_a/K_GO6P_6PGDH_a)*(1+u_nadp_c_a /K_NADP_6PGDH_a)+(1+u_ru5p_D_c_a/K_RU5P_6PGDH_a)*(1+u_nadph_c_a/K_NADPH_6PGDH_a)-1))
RPI_n(u_r5p_c_n,u_ru5p_D_c_n) = 1*(VmaxRPI_n*(1/K_RU5P_RPI_n)*(u_ru5p_D_c_n -u_r5p_c_n/KeqRPI_n)/((1+u_ru5p_D_c_n/K_RU5P_RPI_n)+(1+u_r5p_c_n/K_R5P_RPI_n)-1))

Table U. Julia Fluxes (Part 13)

Fluxes
RPI_a(u_r5p_c_a,u_ru5p_D_c_a) = 1*(VmaxRPI_a*(1/K_RU5P_RPI_a)*(u_ru5p_D_c_a -u_r5p_c_a/KeqRPI_a)/((1+u_ru5p_D_c_a/K_RU5P_RPI_a)+(1+u_r5p_c_a/K_R5P_RPI_a)-1))
RPE_n(u_xu5p_D_c_n,u_ru5p_D_c_n) = 1*(VmaxRPE_n*(1/K_RU5P_RPE_n)*(u_ru5p_D_c_n -u_xu5p_D_c_n/KeqRPE_n)/((1+u_ru5p_D_c_n/K_RU5P_RPE_n)+(1+u_xu5p_D_c_n /K_X5P_RPE_n)-1))
RPE_a(u_ru5p_D_c_a,u_xu5p_D_c_a) = 1*(VmaxRPE_a*(1/K_RU5P_RPE_a)*(u_ru5p_D_c_a -u_xu5p_D_c_a/KeqRPE_a)/((1+u_ru5p_D_c_a/K_RU5P_RPE_a)+(1+u_xu5p_D_c_a /K_X5P_RPE_a)-1))
TKT1_n(u_s7p_c_n,u_r5p_c_n,u_xu5p_D_c_n,u_g3p_c_n) = 1*(VmaxTKL1_n*(1 /(K_X5P_TKL1_n*K_R5P_TKL1_n))*(u_xu5p_D_c_n*u_r5p_c_n-u_g3p_c_n*u_s7p_c_n /KeqTKL1_n)/((1+u_xu5p_D_c_n/K_X5P_TKL1_n)*(1+u_r5p_c_n/K_R5P_TKL1_n)+(1+u_g3p_c_n /K_GAP_TKL1_n)*(1+u_s7p_c_n/K_S7P_TKL1_n)-1))
TKT1_a(u_r5p_c_a,u_g3p_c_a,u_s7p_c_a,u_xu5p_D_c_a) = 1*(VmaxTKL1_a*(1 /(K_X5P_TKL1_a*K_R5P_TKL1_a))*(u_xu5p_D_c_a*u_r5p_c_a-u_g3p_c_a*u_s7p_c_a /KeqTKL1_a)/((1+u_xu5p_D_c_a/K_X5P_TKL1_a)*(1+u_r5p_c_a/K_R5P_TKL1_a)+(1+u_g3p_c_a /K_GAP_TKL1_a)*(1+u_s7p_c_a/K_S7P_TKL1_a)-1))
TKT2_n(u_f6p_c_n,u_e4p_c_n,u_xu5p_D_c_n,u_g3p_c_n) = 1*(VmaxTKL2_n*(1 /(K_F6P_TKL2_n*K_GAP_TKL2_n))*(u_f6p_c_n*u_g3p_c_n-u_xu5p_D_c_n*u_e4p_c_n /KeqTKL2_n)/((1+u_f6p_c_n/K_F6P_TKL2_n)*(1+u_g3p_c_n/K_GAP_TKL2_n)+(1+u_xu5p_D_c_n /K_X5P_TKL2_n)*(1+u_e4p_c_n/K_E4P_TKL2_n)-1))
TKT2_a(u_g3p_c_a,u_e4p_c_a,u_xu5p_D_c_a,u_f6p_c_a) = 1*(VmaxTKL2_a*(1 /(K_F6P_TKL2_a*K_GAP_TKL2_a))*(u_f6p_c_a*u_g3p_c_a-u_xu5p_D_c_a*u_e4p_c_a /KeqTKL2_a)/((1+u_f6p_c_a/K_F6P_TKL2_a)*(1+u_g3p_c_a/K_GAP_TKL2_a)+(1+u_xu5p_D_c_a /K_X5P_TKL2_a)*(1+u_e4p_c_a/K_E4P_TKL2_a)-1))
TALA_n(u_f6p_c_n,u_e4p_c_n,u_g3p_c_n,u_s7p_c_n) = 1*(VmaxTAL_n*(1/(K_GAP_TAL_n *K_S7P_TAL_n))*(u_g3p_c_n*u_s7p_c_n-u_f6p_c_n*u_e4p_c_n/KeqTAL_n)/((1+u_g3p_c_n /K_GAP_TAL_n)*(1+u_s7p_c_n/K_S7P_TAL_n)+(1+u_f6p_c_n/K_F6P_TAL_n)*(1+u_e4p_c_n /K_E4P_TAL_n)-1))
TALA_a(u_g3p_c_a,u_e4p_c_a,u_s7p_c_a,u_f6p_c_a) = 1*(VmaxTAL_a*(1/(K_GAP_TAL_a *K_S7P_TAL_a))*(u_g3p_c_a*u_s7p_c_a-u_f6p_c_a*u_e4p_c_a/KeqTAL_a)/((1+u_g3p_c_a /K_GAP_TAL_a)*(1+u_s7p_c_a/K_S7P_TAL_a)+(1+u_f6p_c_a/K_F6P_TAL_a)*(1+u_e4p_c_a /K_E4P_TAL_a)-1))
notBigg_psiNADPHox_n(u_nadph_c_n) = 1*(k1NADPHox_n*u_nadph_c_n)

Table V. Julia Fluxes (Part 14)

Fluxes
notBigg_psiNADPHox_a(u_nadph_c_a) = 1*(k1NADPHox_a*u_nadph_c_a)
GTHO_n(u_nadph_c_n,u_gthox_c_n) = 1*((Vmf_GSSGR_n*u_gthox_c_n*u_nadph_c_n) /((KmGSSGRGSSG_n+u_gthox_c_n)*(KmGSSGRNADPH_n+u_nadph_c_n)))
GTHO_a(u_gthox_c_a,u_nadph_c_a) = 1*((Vmf_GSSGR_a*u_gthox_c_a*u_nadph_c_a) /((KmGSSGRGSSG_a+u_gthox_c_a)*(KmGSSGRNADPH_a+u_nadph_c_a)))
GTHP_n(u_gthrd_c_n) = 1*(V_GPX_n*u_gthrd_c_n/(u_gthrd_c_n+KmGPXGSH_n))
GTHP_a(u_gthrd_c_a) = 1*(V_GPX_a*u_gthrd_c_a/(u_gthrd_c_a+KmGPXGSH_a))
GTHS_n(u_gthrd_c_n) = 1*(VmaxGSHsyn_n*(glycine_n*glutamylCys_n-u_gthrd_c_n/KeGSHSyn_n) /(Km_glutamylCys_GSHsyn_n*Km_glycine_GSHsyn_n+glutamylCys_n*Km_glutamylCys_GSHsyn_n +glycine_n*Km_glycine_GSHsyn_n*(1+glutamylCys_n/Km_glutamylCys_GSHsyn_n)+u_gthrd_c_n /KmGSHsyn_n))
GTHS_a(u_gthrd_c_a) = 1*(VmaxGSHsyn_a*(glycine_a*glutamylCys_a-u_gthrd_c_a/KeGSHSyn_a) /(Km_glutamylCys_GSHsyn_a*Km_glycine_GSHsyn_a+glutamylCys_a*Km_glutamylCys_GSHsyn_a +glycine_a*Km_glycine_GSHsyn_a*(1+glutamylCys_a/Km_glutamylCys_GSHsyn_a)+u_gthrd_c_a /KmGSHsyn_a))
ADNCYC_a(u_camp_c_a,u_atp_c_a) = 1*(((VmaxfAC_a*u_atp_c_a/(KmACATP_a*(1+u_camp_c_a /KicAMPAC_a))-VmaxrAC_a*u_camp_c_a/(KmpiAC_a*KmcAMPAC_a))/(1+u_atp_c_a/(KmACATP_a*(1 +u_camp_c_a/KicAMPAC_a))+u_camp_c_a/KmcAMPAC_a)))
CKc_n(u_pcreat_c_n,u_atp_c_n,u_adp_c_n) = 1*(kCKnps*u_pcreat_c_n*u_adp_c_n -KeqCKnpms*kCKnps*(Crtot-u_pcreat_c_n)*u_atp_c_n)
CKc_a(u_adp_c_a,u_atp_c_a,u_pcreat_c_a) = 1*(kCKgps*u_pcreat_c_a*u_adp_c_a -KeqCKgpms*kCKgps*(Crtot-u_pcreat_c_a)*u_atp_c_a)

Table W. Julia Fluxes (Part 15)

**Fluxes**
PYRt2m_n(u_h_m_n,u_pyr_m_n,u_h_c_n,u_pyr_c_n) = 1*(mito_scale<br>* Vmax_PYRtrcyt2mito_nH*(u_pyr_c_n*u_h_c_n-u_pyr_m_n*u_h_m_n)/((1.0+u_pyr_c_n<br>/KmPyrCytTr_n)*(1.0+u_pyr_m_n/KmPyrMitoTr_n)))
PYRt2m_a(u_h_m_a,u_pyr_c_a,u_h_c_a,u_pyr_m_a) = 1*mito_scale<br>* (Vmax_PYRtrcyt2mito_aH*(u_pyr_c_a*u_h_c_a-u_pyr_m_a*u_h_m_a)/((1.0+u_pyr_c_a<br>/KmPyrCytTr_a)*(1.0+u_pyr_m_a/KmPyrMitoTr_a)))
notBigg_vShuttlen(u_nadh_m_n,u_nadh_c_n) = 1*mito_scale * (TnNADH_jlv*(u_nadh_c_n<br>/(0.212-u_nadh_c_n))/(MnCyto_jlv+(u_nadh_c_n/(0.212-u_nadh_c_n)))*((1000*NADtot<br>-u_nadh_m_n)/u_nadh_m_n)/(MnMito_jlv+((1000*NADtot-u_nadh_m_n)/u_nadh_m_n)))
notBigg_vShuttleg(u_nadh_c_a,u_nadh_m_a) = 1*mito_scale * (TgNADH_jlv*(u_nadh_c_a<br>/(0.212-u_nadh_c_a))/(MgCyto_jlv+(u_nadh_c_a/(0.212-u_nadh_c_a)))*((1000*NADtot<br>-u_nadh_m_a)/u_nadh_m_a)/(MgMito_jlv+((1000*NADtot-u_nadh_m_a)/u_nadh_m_a)))
notBigg_vMitooutn_n(u_nadh_m_n,u_nad_m_n,u_o2_c_n,u_adp_i_n,u_atp_i_n) = 1<br>*mito_scale * (V_oxphos_n*((1/(u_atp_i_n/u_adp_i_n))/(mu_oxphos_n+(1/(u_atp_i_n<br>/u_adp_i_n))))*((u_nadh_m_n/u_nad_m_n)/(nu_oxphos_n+(u_nadh_m_n/u_nad_m_n)))<br>*(u_o2_c_n/(u_o2_c_n+K_oxphos_n)))
notBigg_vMitooutg_a(u_atp_i_a,u_nadh_m_a,u_nad_m_a,u_o2_c_a,u_adp_i_a) = 1<br>*mito_scale * (V_oxphos_a*((1/(u_atp_i_a/u_adp_i_a))/(mu_oxphos_a+(1/(u_atp_i_a<br>/u_adp_i_a))))*((u_nadh_m_a/u_nad_m_a)/(nu_oxphos_a+(u_nadh_m_a/u_nad_m_a)))<br>*(u_o2_c_a/(u_o2_c_a+K_oxphos_a)))
notBigg_vMitoinn(u_pyr_m_n,u_nadh_m_n) = 1*mito_scale * (VMaxMitoinn*u_pyr_m_n<br>/(u_pyr_m_n+KmMito)*(1000*NADtot-u_nadh_m_n)/(1000*NADtot-u_nadh_m_n+KmNADn_jlv))
notBigg_vMitoing(u_nadh_m_a,u_pyr_m_a) = 1*mito_scale * (VMaxMitoing*u_pyr_m_a<br>/(u_pyr_m_a+KmMito_a)*(1000*NADtot-u_nadh_m_a)/(1000*NADtot-u_nadh_m_a+KmNADg_jlv))

Table X. Julia Fluxes (Part 16)

Julia idx	Variable name	Initial value
1	C_H_mitomatr_n	1.82084728579186e-5 mM
2	K_x_n	54.88513909303926 mM
3	Mg_x_n	0.6405401937956016 mM
4	NADHmito_n	0.3146584474671007 mM
5	QH2mito_n	0.015506712083093229 mM
6	CytCredmito_n	0.13185025457327085 mM
7	O2_n	0.03148650251580875 mM
8	ATPmito_n	0.7487433393982624 mM
9	ADPmito_n	1.8512566606017409 mM
10	ATP_mx_n	0.20845177459661043 mM
11	ADP_mx_n	1.129008031715835 mM
12	Pimito_n	17.22873019667985 mM
13	ATP_i_n	1.385172099080793 mM
14	ADP_i_n	0.057206724603648985 mM
15	AMP_i_n	0.0 mM
16	ATP_mi_n	1.2787221400985913 mM
17	ADP_mi_n	0.05605499445123443 mM
18	Pi_i_n	19.999860977665755 mM
19	MitoMembrPotent_n	152.33918637408712 mV
20	Ctot_n	2.7 mM
21	Qtot_n	1.35 mM
22	C_H_ims_n	3.981071705487114e-5 mM
23	ATP_n	1.3846374147608125 mM
24	ADP_n	0.047784 mM
25	FUMmito_n	0.07033038347789124 mM
26	MALmito_n	0.3877284278881228 mM
27	OXAmito_n	0.01133083263100033 mM
28	SUCmito_n	0.3913984292428137 mM
29	SUCCOAmito_n	0.002765202443496285 mM
30	CoAmito_n	0.0027278355391079485 mM
31	AKGmito_n	0.07302791984472035 mM
32	CaMito_n	0.0001 mM
33	ISOCITmito_n	0.03312781257435126 mM
34	CITmito_n	0.3414510962627293 mM
35	AcCoAmito_n	0.040486317761993094 mM
36	AcAc_n	0.0008938613668892289 mM
37	AcAcCoA_n	3.471725572540322e-5 mM
38	PYRmito_n	0.04592802047818836 mM
39	bHB_n	0.002050830230641291 mM
40	bHB_ecs	0.002861722937272813 mM
41	bHB_a	0.002235 mM
42	bHB_b	0.2981203841045341 mM
43	ASPmito_n	1.4 mM
44	ASP_n	1.4 mM
45	GLUmito_n	10.001887920296111 mM
46	MAL_n	0.45 mM
47	OXA_n	0.01 mM
48	AKG_n	0.2 mM
49	GLU_n	10.000000420931526 mM
50	NADH_n	0.004140780436510287 mM

Table Y. Initial values of metabolism variables.

Julia idx	Variable name	Initial value
51	C_H_mitomatr_a	1.82084728579186e-5 mM
52	K_x_a	54.88513909303926 mM
53	Mg_x_a	0.6254020744170418 mM
54	NADHmito_a	0.3622495632039702 mM
55	QH2mito_a	0.010658928492490746 mM
56	CytCredmito_a	0.10755014489041778 mM
57	O2_a	0.03905865747239789 mM
58	ATPmito_a	0.8554395319513812 mM
59	ADPmito_a	1.7445604680486215 mM
60	ATP_mx_a	0.20827897454727567 mM
61	ADP_mx_a	1.1443189511420615 mM
62	Pimito_a	17.625288473162165 mM
63	ATP_i_a	1.29745793823728 mM
64	ADP_i_a	0.044346699541431896 mM
65	AMP_i_a	0.0 mM
66	ATP_mi_a	1.1823280190768068 mM
67	ADP_mi_a	0.04362949589266071 mM
68	Pi_i_a	19.999931107524688 mM
69	MitoMembrPotent_a	155.39201516889315 mV
70	Ctot_a	2.7 mM
71	Qtot_a	1.35 mM
72	C_H_ims_a	3.981071705487114e-5 mM
73	ATP_a	1.2971907639974427 mM
74	ADP_a	0.045 mM
75	FUMmito_a	0.05005917725596374 mM
76	MALmito_a	0.25524996956098295 mM
77	OXAmito_a	0.004468226870983686 mM
78	SUCmito_a	0.5865999694835419 mM
79	SUCCOAmito_a	0.0016699868314113365 mM
80	CoAmito_a	0.002541368839174815 mM
81	AKGmito_a	0.015159157992632254 mM
82	CaMito_a	0.0001 mM
83	ISOCITmito_a	0.0360692382798456 mM
84	CITmito_a	0.358985824746709 mM
85	AcCoAmito_a	0.004288644329412937 mM
86	AcAc_a	0.00312 mM
87	AcAcCoA_a	0.0006 mM
88	PYRmito_a	0.012983253151168756 mM
89	GLN_n	0.40016780751134395 mM
90	GLN_out	0.19991032677727677 mM
91	GLN_a	0.24979819268840564 mM
92	GLUT_a	0.2994675379035365 mM
93	Va	-94.73739998434871 mV
94	Na_a	16.27886560396611 mM
95	K_a	100.79307843484172 mM
96	K_out	3.1104211426858464 mM
97	GLUT_syn	2.5e-5 mM
98	VNeu	-68.00932293426199 mV
99	Na_n	8.473759755301563 mM
100	h	0.9811237297560305

Table Z. Initial values of metabolism variables.

Julia idx	Variable Name	Initial Value
101	n	0.03467357454611763
102	Ca_n	5.436689205733492e-5 mM
103	pgate	0.03553923881632591
104	nBK_a	7.45614720951229e-6 mM
105	mGluRboundRatio_a	0.0 mM
106	IP3_a	1.1852340589842885e-6 mM
107	hIP3Ca_a	0.6531565346093099 mM
108	Ca_a	5.31000955574647e-5 mM
109	Ca_r_a	0.4 mM
110	sTRP_a	0.00124697236672799
111	vV	0.02370000249731734 ml
112	EET_a	3.143871670033718e-5 mM
113	ddHb	0.05852770291944967 mM
114	O2cap	7.105220869902977 mM
115	Glc_b	4.555436497117541 mM
116	Glc_t_t	1.4036921120261527 mM
117	Glc_ecsBA	1.302331013714969 mM
118	Glc_a	1.2215129412034003 mM
119	Glc_ecsAN	1.0034276408975709 mM
120	Glc_n	0.9117230433328605 mM
121	G6P_n	0.04518031109219087 mM
122	G6P_a	0.06142242564901545 mM
123	F6P_n	0.007367188108993513 mM
124	F6P_a	0.010139153003705685 mM
125	FBP_n	0.009458862178707486 mM
126	FBP_a	0.029976744727305948 mM
127	f26bp_a	0.012169896922510802 mM
128	GLY_a	13.99969995417289 mM
129	AMP_n	1.0e-5 mM
130	AMP_a	1.0e-5 mM
131	G1P_a	0.012811280361731594 mM
132	GAP_n	0.0012351454067088853 mM
133	GAP_a	0.0022568560606488503 mM
134	DHAP_n	0.003220803533622665 mM
135	DHAP_a	0.0037897580104066415 mM
136	BPG13_n	0.0060805407271592475 mM
137	BPG13_a	0.008030562619956915 mM
138	NADH_a	0.10927809092221007 mM
139	Pi_n	20.0 mM
140	Pi_a	20.0 mM
141	PG3_n	0.01765067153478949 mM
142	PG3_a	0.021463604134103274 mM
143	PG2_n	0.00196123874550032 mM
144	PG2_a	0.0023600313501276823 mM
145	PEP_n	0.0011299114036925676 mM
146	PEP_a	0.0013498159787353333 mM
147	Pyr_n	0.09884562894707946 mM
148	Pyr_a	0.029482442303259512 mM
149	Lac_b	0.6887631395085969 mM
150	Lac_ecs	0.6223377821910432 mM

Table AA. Initial values of metabolism variables.

Julia idx	Variable Name	Initial Value
151	Lac_a	0.6224222941358745 mM
152	Lac_n	0.62189156291357 mM
153	NADPH_n	0.030030028188024867 mM
154	NADPH_a	0.03000617948372434 mM
155	GL6P_n	2.7223858669789458e-6 mM
156	GL6P_a	3.019823774010181e-6 mM
157	GO6P_n	0.0026154588421574165 mM
158	GO6P_a	0.001825222802612153 mM
159	RU5P_n	0.0005499402428921933 mM
160	RU5P_a	0.0006682285740983806 mM
161	R5P_n	1.564984868864245e-5 mM
162	R5P_a	2.6065883215538542e-5 mM
163	X5P_n	0.016834424262979618 mM
164	X5P_a	0.020470510408354833 mM
165	S7P_n	0.019807839436742886 mM
166	S7P_a	0.24954311556796605 mM
167	E4P_n	0.006632954456360935 mM
168	E4P_a	0.006138869748662782 mM
169	GSH_n	1.200006238770546 mM
170	GSH_a	4.300000202932421 mM
171	GSSG_n	0.0119968806147281 mM
172	GSSG_a	0.04299989853382339 mM
173	Cr_n	5.053977657681814 mM
174	PCr_n	4.98986852470732 mM
175	Cr_a	5.0757940916174356 mM
176	PCr_a	4.927691727614907 mM
177	cAMP_a	0.039182703258954135 mM
178	NE_neuromod	0.0 mM
179	UDPgluco_a	0.109 mM
180	UTP_a	0.23 mM
181	GS_a	0.0029999 mM
182	GPa_a	0.0013314628973952977 mM
183	GPb_a	0.06866853710260439 mM

Table AB. Initial values of metabolism variables.
