# Supplementary material for: A multiscale electro-metabolic model of a rat neocortical circuit reveals the impact of ageing on central cortical layers
Source: PLoS Comput Biol. 2025 May 20;21(5):e1013070. doi: 10.1371/journal.pcbi.1013070 (PMC12112163; doi:10.1371/journal.pcbi.1013070)
Supplement: S3 Text — Fig A: ATP production and consumption per e-type. Fig B: Dunn test heatmap corresponding to Fig 4. Table A: ATP production and consumption per e-type. Table B: Correlation between ATP consumption and production per e-type. Table C: Statistical analysis of ATP/s consumption across neural layers. Table D: Descriptive statistics across layers for resting membrane potential, spike count, maximum voltage, and action potential (AP) amplitude. Table E: Descriptive statistics across e-types for resting membrane potential, spike count, maximum voltage, and action potential (AP) amplitude. Table F: Comparison of total spike count and average ATP consumption. (PDF) [file pcbi.1013070.s004.pdf]

S3 Text: A multiscale electro-metabolic model of a rat neocortical circuit reveals the impact of ageing on central cortical layers

Sofia Farina[1], Alessandro Cattabiani[1], Darshan Mandge[1], Polina Shichkova[1,2], James B. Isbister[1], Jean Jacquemier[1], James G. King[1], Henry Markram[1,3], Daniel Keller[1]

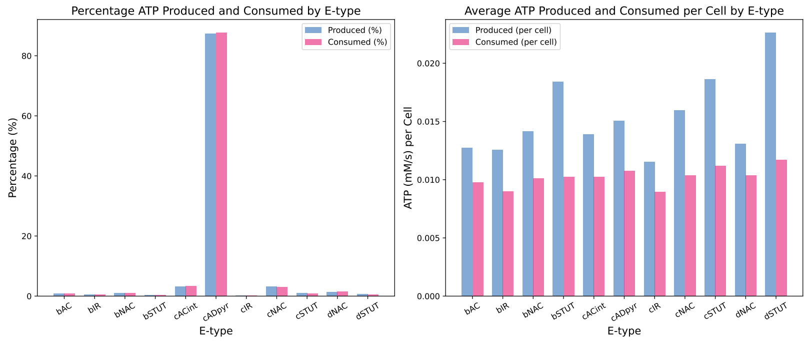

Fig A. ATP **production and consumption per e-type.** Statistical analysis of ATP production and consumption averaged over the simulation per e-type. It is related to Fig 2D of the manuscript.

**E-type**	ATP **Prod. (%)**	ATP **Cons. (%)**	ATP **Prod. (mM/s)**	ATP **Cons. (mM/s)**
bAC	0.876	0.943	0.0128	0.0098
bIR	0.502	0.504	0.0126	0.0090
bNAC	1.026	1.028	0.0141	0.0101
bSTUT	0.434	0.339	0.0184	0.0103
cACint	3.207	3.310	0.0139	0.0102
cADpyr	87.325	87.684	0.0151	0.0108
cIR	0.225	0.245	0.0115	0.0089
cNAC	3.281	3.000	0.0160	0.0104
cSTUT	0.989	0.834	0.0186	0.0112
dNAC	1.456	1.620	0.0131	0.0104
dSTUT	0.678	0.493	0.0226	0.0117

Table A. ATP **production and consumption per e-type.** Statistical analysis of ATP production and consumption averaged over the simulation per e-type. It is related to Fig 2D of the manuscript.

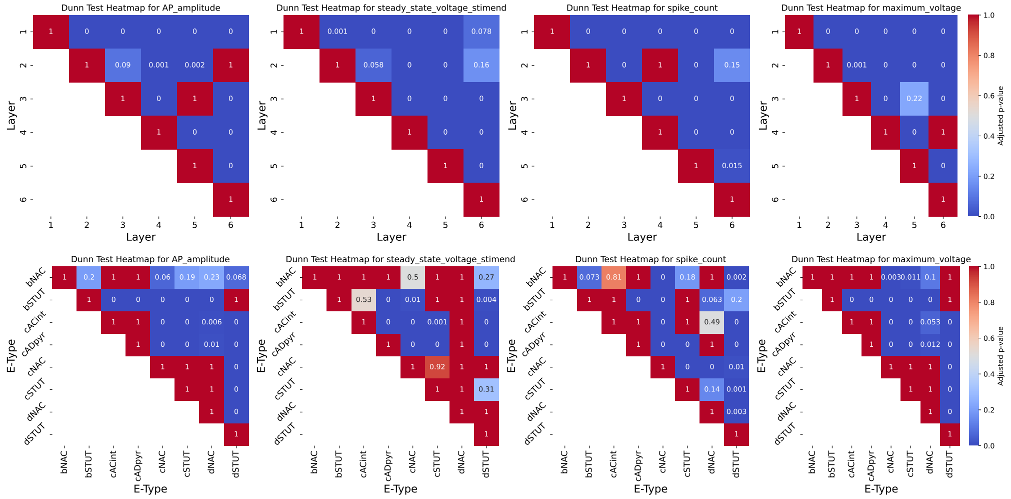

Fig B. Dunn test heatmap corresponding to Fig 4.

Group	Pearson Corr.	p-value	Spearman Corr.	p-value
bAC	0.96	7.91e-158	0.98	6.00e-190
bIR	0.96	8.62e-92	0.96	2.23e-98
bNAC	0.97	2.31e-190	0.97	1.39e-196
bSTUT	0.94	1.54e-48	0.97	9.80e-59
cACint	0.96	0.00	0.97	0.00
cADpyr	0.96	0.00	0.97	0.00
cIR	0.97	3.97e-50	0.97	1.41e-53
cNAC	0.91	0.00	0.95	0.00
cSTUT	0.93	1.13e-96	0.95	1.59e-114
dNAC	0.97	2.13e-300	0.98	7.99e-321
dSTUT	0.91	1.78e-48	0.94	1.44e-58

Table B. Correlation between ATP **consumption and production per e-type.** Pearson and Spearman correlation coefficients and p-values were calculated for the average ATP consumed and produced throughout the simulation per e-type. This is related to Fig 3A of the manuscript.

Layer	AP mean	AP std	AP median	Rest mean	Rest std	Rest median	T-test pval
1	4.64e+08	2.16e+08	4.10e+08	2.24e+08	1.05e+08	2.04e+08	4.65e-17
2	5.10e+08	3.16e+08	4.13e+08	2.13e+08	1.11e+08	1.95e+08	9.53e-24
3	4.75e+08	2.68e+08	4.15e+08	1.80e+08	7.70e+07	1.70e+08	2.52e-76
4	4.67e+08	2.69e+08	3.95e+08	2.25e+08	9.53e+07	2.07e+08	4.59e-160
5	4.66e+08	2.57e+08	4.07e+08	2.11e+08	9.21e+07	1.97e+08	5.21e-284
6	4.77e+08	2.62e+08	4.16e+08	2.02e+08	8.26e+07	1.88e+08	5.16e-103

Table C. Statistical analysis of ATP/s **consumption across neural layers.** Mean, standard deviation, and median values of ATP/s consumption at rest and during action potentials (APs) are summarized, as shown in Fig 3C. t-test p-values comparing ATP/s consumption between AP and resting states across different layers are also reported.

		**Resting State** (mV)		**Spike Count**		**Max Voltage** (mV)		**AP Amplitude** (mV)	
**Layer**	**# Sample**	**Mean**	**Std**	**Mean**	**Std**	**Mean**	**Std**	**Mean**	**Std**
1	165	-75.74	2.99	5.05	2.40	-0.57	8.02	64.72	7.27
2	437	-72.85	4.22	4.15	4.63	15.39	12.10	75.35	9.44
3	1277	-70.66	1.94	1.90	2.04	20.67	5.40	78.33	4.41
4	1963	-69.11	2.58	2.75	2.12	13.01	9.11	72.84	8.74
5	639	-68.66	4.54	4.59	3.77	21.32	10.05	78.56	9.06
6	165	-73.47	3.36	3.45	2.57	14.16	8.22	75.97	7.15

Table D. Descriptive statistics across layers for resting membrane potential, spike count, maximum voltage, and action potential (AP) amplitude. Statistics are shown as related to Fig 4 of the manuscript.

		Resting State (mV)		Spike Count		Max Voltage (mV)		AP Amplitude (mV)	
**Layer**	**# Sample**	**Mean**	**Std**	**Mean**	**Std**	**Mean**	**Std**	**Mean**	**Std**
bNAC	6	-70.28	1.61	1.50	1.22	20.42	3.07	77.46	4.86
bSTUT	75	-74.16	4.20	4.09	2.09	24.05	4.05	84.23	3.73
cACint	52	-70.98	2.65	4.17	3.66	18.08	6.26	78.81	7.16
cADpyr	3886	-68.83	2.92	3.17	2.74	18.82	7.86	76.98	6.71
cNAC	304	-77.16	3.17	9.12	4.60	-1.17	8.88	63.02	8.08
cSTUT	142	-75.68	4.73	3.59	1.75	0.64	11.43	63.65	10.65
dNAC	4	-71.05	1.32	1.00	0.00	-10.06	6.94	48.41	6.18
dSTUT	105	-79.09	4.24	5.64	2.56	31.84	13.75	95.69	13.33

Table E. Descriptive statistics across e-types for resting membrane potential, spike count, maximum voltage, and action potential (AP) amplitude. Statistics are shown as related to Fig 4 of the manuscript.

**Simulation**	**Total Spike Sum**	**Average** ATP mM
Neurodamus	14,640	1.38
Young	16,893	1.18
Aged	17,135	1.11

Table F. Comparison of total spike count and average ATP **consumption.** Three simulation scenarios are compared: Neurodamus (electrophysiological model with constant ATP), Young, and Aged.
