# Supplementary material for: A multiscale electro-metabolic model of a rat neocortical circuit reveals the impact of ageing on central cortical layers
Source: PLoS Comput Biol. 2025 May 20;21(5):e1013070. doi: 10.1371/journal.pcbi.1013070 (PMC12112163; doi:10.1371/journal.pcbi.1013070)
Supplement: Attachment — Submitted filename: ResponsetoReviewers.pdf [file pcbi.1013070.s005.pdf]

Dear Professor Jonhatan Rubin and Professor Hugues Berry,

Dear Reviewers,

Thank you for the evaluation of our manuscript and the constructive comments
Based on the feedback, we have adapted and extended the manuscript to address the questions and comments.

Please find below the point-to-point responses and explanation to the questions and remarks made by the reviewers to the manuscript.

Response to the reviewers

Detailed Responses to Questions.

Reviewer #1: The manuscript presents an innovative computational framework combining electrophysiology and metabolism in a reconstructed rat neocortical circuit. The work provides an in-depth analysis of the energy demands of neuronal activity by layers and specific cell types, and provides interesting insights such as how metabolic aging impacts more strongly central cortical layers. This study is particularly noteworthy at the technical level, for its integration of a highly detailed electrophysiological model of cortical circuits with a complex and validated neuro-glia-vascular metabolic model.
While the work is novel and a useful contribution to the field, there are a number of issues that need to be addressed to ensure clarity and validity of the conclusions.

All results appear to rely on two 3-sec simulations, one with constant ATP level and one with the metabolic process. It is therefore unclear if the results are valid for different initial conditions / randomization, for longer durations, and for different states/conditions (e.g. with sensory inputs instead of spontaneous conditions). More than one simulation per condition would be required to quantify results statistically, ensuring they are robust and the metabolic model is not overfitted. Additionally, a longer simulation would demonstrate the results remain valid beyond 3 seconds.

We appreciate the thoughtful feedback regarding the need to validate our results across different initial conditions, randomization seeds, simulation durations, and input conditions. Many of these important aspects have already been addressed in previous studies, in which the individual models were originally developed and rigorously tested before being coupled. Given the high computational demands of the full circuit simulation, we focused on integrating these well-validated components rather than redoing extensive standalone testing.

Electrophysiological model validation
In the electrical component of the circuit (a single neuron), each of the 40 e-models was optimized for different step-depolarizing and hyperpolarizing stimulus protocols, as described in Reva et al., 2024. The initial ionic concentrations used are noted in the Section 4 from S1 Text.

These e-models were then generalized for approximately 129k neurons. As an example, the layer 5 pyramidal neuron e-model, cADPyr_L5TPC, uses over 10 protocols: depolarizing steps (150%, 200%, 280%, 320% of rheobase current), hyperpolarizing steps (-100% and -40%), a protocol for backpropagating action potentials, a protocol for input resistance, and resting membrane potentials (see **Reviewer Response Figure 1**).

We also validated our single-cell model with stimuli not used during optimization to assess the strength of the selected e-model. Additionally, we optimized all ionic concentrations, including ATP, to ensure that values remained within physiological bounds. The e-model optimization parameters, such as conductances and pump rates, were either tuned to be within the bounds described in previous experimentally validated models, or defined to be within the bounds suggested by experimental data. We performed several optimization iterations (seeds) and selected the one with the best performance for all the above protocols. We believe doing so prevents overfitting of models and ensures that the selected models, along with other circuit components, will perform well.

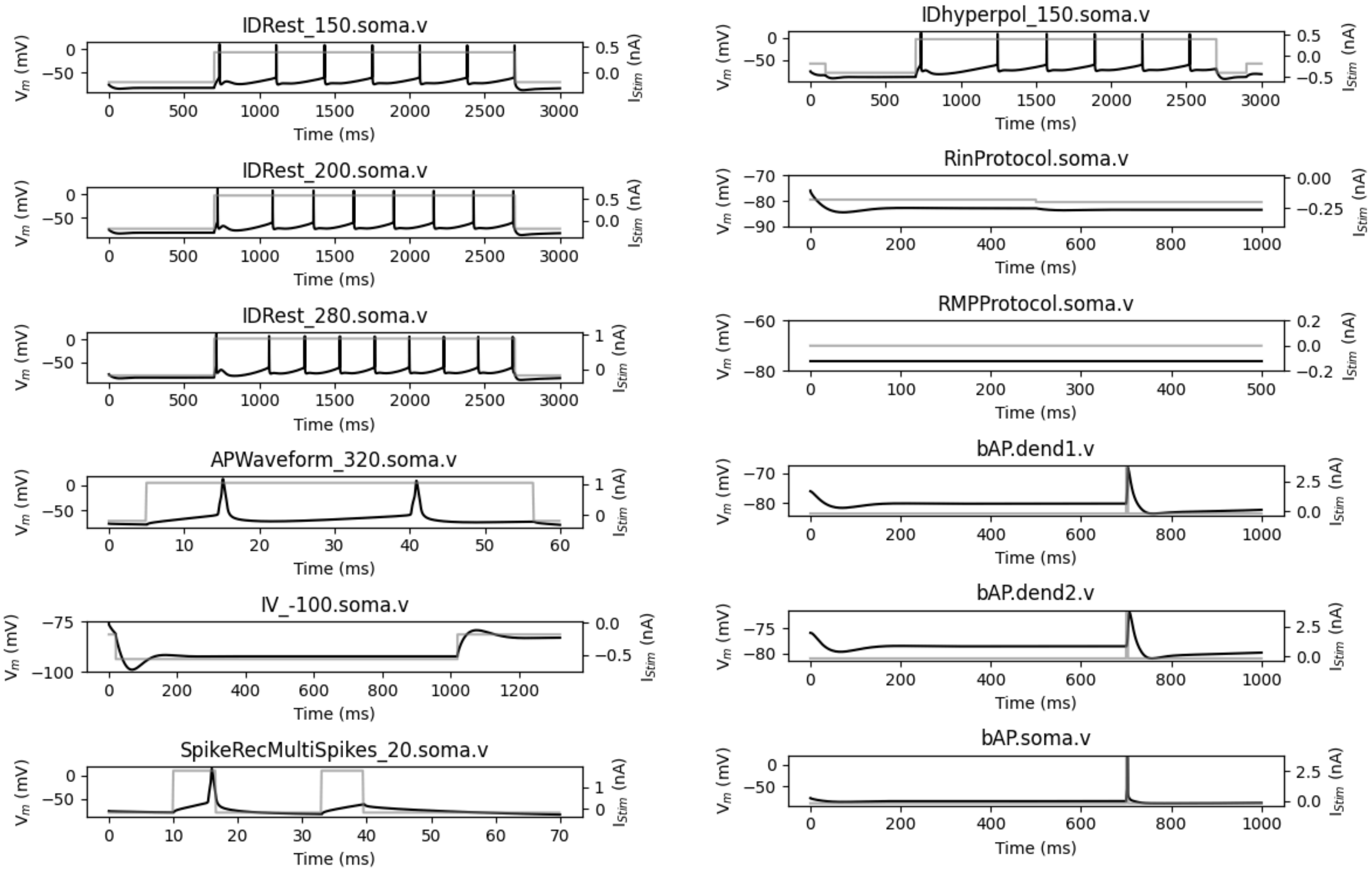

Reviewer Response Figure 1: cADpyr_L5TPC e-model optimisation protocol responses for testing the robustness of the e-model generated. The dark lines are responses of the e-model to the current stimulus injections in the neuron soma (grey lines). We used several depolarising (IDRest, APWaveform, SpikeRec), hyperpolarising (IV), input resistance (RinProtocol) and backpropagating action potential (bAP) protocols for the e-model. We also optimized for

bAP voltage responses on apical dendrite at 208 um (bAP.dend1.v) and 402 um (bAP.dend1.v). We used such diverse stimuli for all the e-models and thus, which endowed the e-models with robustness when used in the whole circuit.

Metabolic model validation

The metabolic model was originally developed as a standalone framework, capturing the intricate metabolism-electrophysiology-blood flow interactions within the NGV unit (Shichkova et al., 2025, in press). This model has been extensively tested across both young and aged conditions, using various stimuli such as current injections and wave-function-modeled synaptic inputs. A thorough analysis of the model's behavior was conducted, including a sensitivity study where each parameter (except for physical constants) was systematically perturbed to assess its impact. The initial conditions were carefully selected based on an extensive review of the literature, ensuring biological realism. The iterative process of model building and optimization is described in detail in that work, providing a strong foundation for our current study.

Choice of simulation duration

Our initial choice of 3-second simulations was driven by computational constraints while still allowing us to capture transient metabolic dynamics and achieve in vivo firing rates. To further evaluate the model's stability, we ran a small-scale simulation (17 neurons) for 10 seconds with the same input stimuli used in the article.

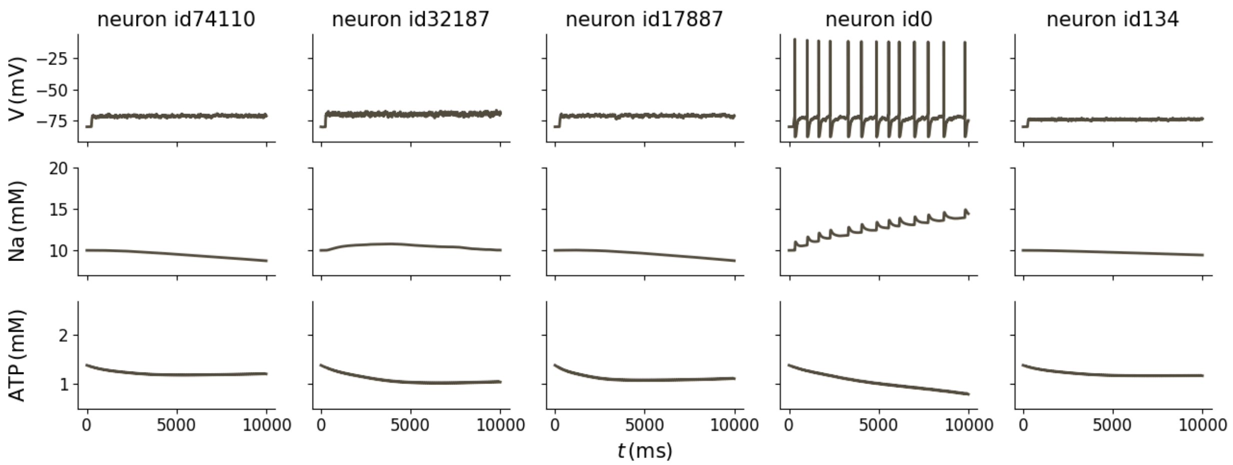

Reviewer Response Figure 2: For the purpose of testing we used a small circuit composed of 17 neurons from different layers and e-types, extracted from the larger circuit. This was employed to test changes in the code, assess the numerical coupling, and verify that our model would behave correctly during simulations longer than 3 seconds. The stimuli applied followed the same protocol used in the main study: there was no stimulus for the first 250 ms, and in-vivo-like stimuli started at 250 ms. Additionally, the circuit was initially disconnected (no synapses) and was connected at 1000 ms.

Alternative input conditions

At the circuit level, simulations were conducted following the protocol of Isbister et al. 2023 with fixed ATP concentrations to identify the layer-specific parameters. We then applied different stimulus conditions to replicate in vivo firing rates.

While our goal in this study was to replicate in vivo firing rates, alternative stimuli could be applied to the circuit or specific subsets of neurons. However, these stimuli must be carefully calibrated to avoid excessive spike bursting and ATP depletion. That said, it is straightforward to change the input stimuli in Neurodamus. **Reviewer Response Figure 3** shows an example of two simulations obtained following the workflow of Isbister et al. (2023) to find the in vivo calibration.

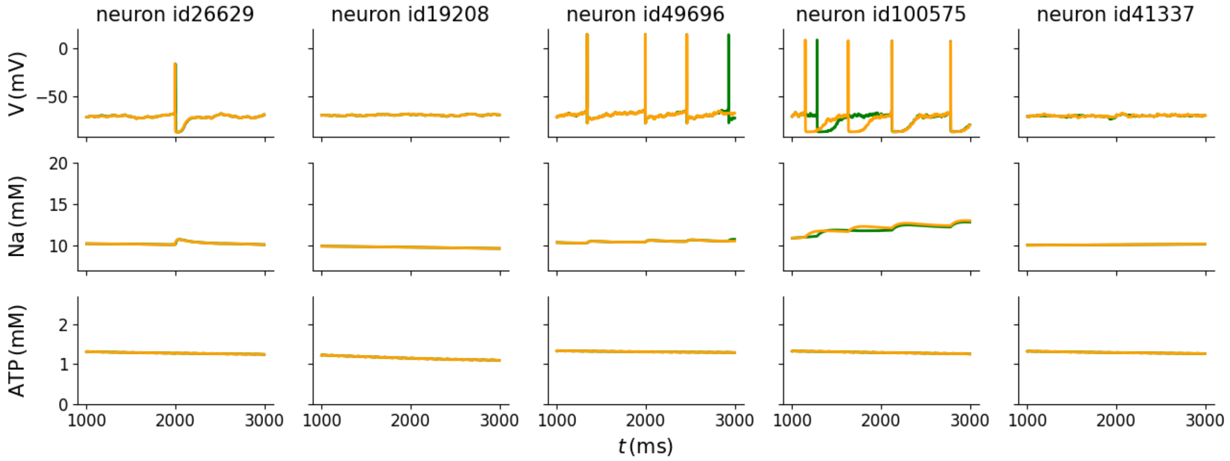

Reviewer Response Figure 3: Following the parameter calibration of Isbister et al., we obtained a range of possible values with two possible calcium levels. Here, we compare the simulation presented in the article with one that had the same input stimuli but different levels of calcium, 1.1 mM (as in the article) versus 1.05 mM (represented by the green line) in this simulation.

Overall, we believe that the robustness and validation of the original models, combined with the developments made in this work, provide a solid foundation as a first step. Further development may involve testing different input stimuli to reproduce in vivo experiments, or investigating additional parameters and randomization. However, this is beyond the scope of the present manuscript and will be investigated in more detail in follow-up studies.

Following this discussion, we have added further details in the Model Validation Section L. 141-145 and L. 152-154.

"Layer-specific mitochondrial densities" (Line 109) - Clarify what other layer-specific data was included. Is there any layer-specific data related to the astrocyte morphologies or spatial distribution? This seems relevant given the layer-specific model predictions.

We thank the reviewer for this remark and we have added clarification to the manuscript. In our model, several layer-specific parameters are incorporated:

- **Neuronal densities**: The circuit construction accounts for natural neuronal densities per layer, ensuring proper calibration of neuron numbers. We clarify that in the Model overview adding L.77-78
- **Electrophysiological properties**: The model is calibrated to reflect layer-specific electrical characteristics. The pyramidal neuron e-models for layers 2/3, 4, 5 and 6 were

optimized with electrical features extracted from respective layer pyramidal neurons. Moreover, layer-specific e-models were created by using reconstructed or cloned morphologies from each cortical layer for both pyramidal (EXC) and inhibitory (INH) neurons. Section 1 Table in S1 Text shows different layer m-types used in the circuit. Such layer-specific electrical and morphological properties impart biological realism to the circuit. We have added an explanation in the model overview: L. 125-130

- **Metabolic modeling**: The only layer-specific metabolic parameter explicitly included is mitochondrial density.
- **Astrocytic morphologies:** Regarding astrocytes, our microcircuit construction follows the approach of Zisis et al. (2021). In the circuit data, astrocytic morphologies are present and positioned based on biological data. We have clarified this point in the Model Overview section: L120-121 Furthermore, we have clarified in the Discussion section that astrocytic morphologies are present in the circuit. As a next step, we propose incorporating these morphological data into a geometry-based metabolic model. L. 409-411

While the results in Fig 5 are interesting, they should be better tied to the rest of the paper; currently, it is not clear why they are relevant and how they relate to the metabolic story.

We appreciate the suggestion. Figure 5 links neuronal morphologies with electrical properties, a relationship made possible by the detailed model we developed, further extending the layer-specific investigation introduced in Figure 4. To better highlight this connection, we added a sentence in L. 264-268. Additionally, to ensure a more comprehensive integration with the rest of the paper, we included updates at the end of the results section (L. 291-294) and in the discussion section (L. 412-414).

"Inherent limitations" (line 394) - Please describe these limitations, as this is an important consideration for future model users. Additionally, given several important model limitations previously described in the Discussion, it would be important to clarify which simulation results have been validated against experimental data and which are predictions to be tested.

Limitations for e-models:
We optimized e-models using somatic patch-clamp recordings, with some additional data from dendritic and axon initial segment recordings available in the literature.
We generalized 40 e-models to multiple m-types, which may not always yield the most accurate responses. This was done by applying the final parameters of the e-models to all corresponding me-model combinations. However, this strategy represents a necessary computational compromise, as optimizing all 129k neurons individually would be infeasible.

Metabolic model limitations:
Due to limited available data, we had to refine weakly constrained parameters and modelled only the most relevant pathways and processes rather than simulating metabolic dynamics at a genome-wide scale.

We did not account for differences between in vitro and in vivo conditions, nor did we consider sex-related differences.
Changes in oxygen availability and transport with aging were also not included due to data limitations.
To derive the scaling of enzyme and transporter concentrations in the aging group, we relied on RNA-level data rather than protein levels, based on the assumption that changes in RNA directly reflect changes in enzyme concentrations (Edfors et al., 2016).
Scaling for the NAD pool and synaptic glutamate release was also literature-driven but had to be approximated, as exact values for their changes with aging were unavailable.

Regarding these limitations, we have expanded the Discussion by adding L. 439-444.

The Metabolism model in the Methods is not described in enough detail, despite its key role in this study. It would be useful to the reader to see a diagram of the model illustrating the different cellular processes and compartments ("neuronal and astrocytic cytosol, mitochondrial matrix and intermembrane space, interstitium, basal lamina, endothelium, capillaries, arteries, and the endoplasmic reticulum.") The supplementary section with the long list of equations does not help to illustrate the basics of how this model works, so to the reader it remains somewhat as a black box model.

We appreciate the feedback and recognize that a visual representation would make the model more accessible to readers. To address this, we have added a schematic diagram of the model as **Figure 1 in Supplementary Text 2** (before the list of equations and fluxes). This diagram provides an overview of the different cellular processes and compartments, helping readers understand the model structure without needing to refer directly to the equations or the original paper (Shichkova et al., 2025). Additionally, we have cited it in the manuscript at L. 119 to improve clarity.

"the energetic demands of the brain account for two-thirds of the body's energy production" (Line 4): Is this correct? The value I commonly see in the literature is 20%.

The sentence has been corrected. The reference originally contained an error and should indicate that the brain uses about two-thirds of the ATP it produces. To avoid misunderstanding we corrected the sentence L4-5, referencing Attwell and Laughlin 2001.

Fig 1 Panel A is potentially misleading as the vasculature and astrocytes are not modeled spatially in 3D. The caption should make it very clear that this is just a depiction, but the model does not include 3D astrocyte or blood vessel morphologies.

The circuit model includes neuronal, astrocytic morphologies, and vasculature. Therefore, the image is not misleading regarding the presence of these components. However, we do not

incorporate the astrocytic morphologies and vasculature in the NGV model solution. We have clarified this by updating the caption of Figure 1.

Fig 2: The produced vs consumed colors differ in the legend vs caption.

Corrected.

Fig 6C: The x-axis description is unclear. Is it the number of neurons or spikes?

The x-axis represents spikes, not neurons. The original caption was unclear, so we have revised it to improve clarity in Figure 6.

"NGV unit and solved it for each neuron" (Line 329): Can NGV units be shared among multiple neurons? or is there a 1 to 1 correspondence? Is this realistic?

Biologically, neurons do not have a strict one-to-one correspondence with NGV units. Astrocytes, which number approximately one-third of neurons in mice, support multiple neurons metabolically, and nearly every neuron is estimated to have its own capillary (Zlokovic 2005). While our model currently solves the metabolic interactions per neuron, a more biologically realistic approach would involve solving the model for both neurons and astrocytes in the circuit. This would allow astrocytes to support multiple neurons, but it would be computationally demanding. Although this is a promising future direction, we believe our current model provides a good approximation while maintaining computational feasibility, as it is common in the literature to focus on a single neuron-glial-vasculature unit.

We have added a sentence in the discussion (L. 406-409) to acknowledge this consideration and highlight potential future directions for improving biological realism in our model.

Reviewer #2: Farina et al. develops a multiscale neuro-glial-vascular model that integrates the electrophysiology of neurons with the metabolic processes. The model is multiscale in time as the fast dynamics of the neuronal electrical activity is coupled with the slower metabolic processes. Another key feature of the model is that the network and morphologies of neuronal models are based on reconstructed rat neocortex. That is, individual neurons are modeled spatially based on their reconstructed morphologies and the network is built on the experimentally observed synaptic densities. The spatial distribution of currents and fluxes follow the observed patterns as well. The model effectively captures electro-metabolic processes at the circuit level, and highlights the importance of heterogeneity within the circuit, where energetic demands vary according to neuronal characteristics. Finally, in metabolic ageing, the model indicates that the middle cortical layers are particularly vulnerable to energy impairment.

Overall, it is an interesting study that provides a valuable platform for investigating neurovascular coupling and the (im)balance between energy demand and supply under different (patho)physiological brain functions. The manuscript and associated code include all details about the model, methods, and results. While normally, I would ask for including more details about the equations used in such complex model and why different equations are chosen, the

fact that the authors have provided all equations in Julia format in the manuscript and the code is archived on github sidesteps the concerns about the reproducibility of the results or reusability of the model.

I do not have any major concerns about this manuscript, and recommend the publication of the paper with a few minor edits.

Line 4-5: “Despite its small size, the energetic demands of the brain are remarkably high, accounting for two-thirds of the body’s energy production [1–4].” Please confirm this statement and mention the conditions under which the brain accounts for two-thirds of the energy demand.

Answered to reviewer 1.

Line 86-88: “To address these differences, the two systems were solved independently in an interleaved manner and synchronized every 100 ms.” This is one part of the model that needs more explanation. It is not clear how exactly was the coupling performed at the time step of updating the two systems (tcoupling) and how were the fast variables tracked between the two consecutive larger timesteps at which the coupling was performed (between tcoupling_i and tcoupling_i+1). I am sure it can be figured out from the code, but it’s a key point and researchers might want to use this approach in other models as well. So, explaining this in the manuscript would make it easier.

Thank you for your insightful comment. We recognize that the time coupling mechanism required further clarification in the Model Overview section. To address this, we have revised the paragraph (L. 83-103) to better explain this key point. Additionally, in response to feedback from Reviewer 3, we have modified Figure 1C, which we believe enhances the overall understanding of the time coupling mechanism.

Line 180: “Synaptic glutamate release pools were also adjusted to 280 account for ageing”. What “adjusted” means? Increased/decreased etc.?

We have corrected that sentence to provide a clearer explanation (L. 309).

Reviewer #3: The authors propose a novel multiscale model of electro-metabolic coupling in the rat neocortex. Their unique approach includes highly detailed morphologies and various electrical types. The electro-metabolic model is then tested under different scenarios such as resting state, activation and metabolic ageing. The results fully agree with the previous literature, showing that the regions of the brain that are characterised by large synaptic densities are the first to be affected by metabolic ageing. While many simplifying assumptions are used within this model, this work is highly ambitious and could serve as a perfect tool for further development in this novel integrative research area.

Questions and Suggestions:

1. The manuscript is very well written, and every single component of the model is extremely

well described. I have found only two typos: legend of Fig 6, panel B (VVisualization) and the second paragraph in the Discussion (Fig.2)-Fig.3).

We thank the reviewer for pointing out the typos, and we have corrected them.

2. The illustration of the time-coupling is a bit confusing and overly simplified. The time-coupling concept became very clear towards the end of the manuscript, once I read the Multiscale Orchestrator. However, I believe the time-coupling is a core idea in this manuscript and it would be nice for the general readers to understand the concept earlier on. I was wondering whether you could modify fig1C to be more meaningful. I think adding a scheme following the orchestration protocol defined on page 17 would be much more understandable.

We agree that the time-coupling mechanism is a core aspect of our manuscript and should be clearly conveyed early on. To address this, we have revised the paragraph on time coupling in the Model Overview section (L. 83-103) for improved clarity. Additionally, we have modified Figure 1C to make the time-coupling concept more intuitive for general readers.

3. Throughout the manuscript you repeat that each metabolic step is 100ms and I understood on page 17 that this choice was done by using the Rosenbrock23 solver. Until I reached page 17, I kept wondering how you selected this value. I wonder if you could comment on what would happen if you selected a smaller/larger step. No additional simulations are needed, only a comment.

Selecting an appropriate coupling time between the metabolic and electrophysiological models is critical for ensuring both biological realism and computational efficiency. The metabolic model generates ATP over a coupling interval [tstart, tcoupling], which is solved using the Rosenbrock23 ODE solver (this should now be clearer following the changes made in response to Reviewer 2, L. 83-104, as well as the updates in Figure 1C). In contrast, the electrophysiological model rapidly consumes ATP through Na-K pump activity.

If the coupling time is too long (e.g., 500 ms), the metabolic system might reach a steady state with maximal ATP production, but the produced ATP remains unused within the system, which is not realistic. Meanwhile, the electrophysiological model depletes its available ATP, leading to biologically unrealistic conditions where ATP concentrations drop to zero. Conversely, an excessively short coupling time (less than 100 ms) does not allow the Rosenbrock solver to take sufficient time steps (Δt) for metabolism to converge toward a solution, preventing sufficient and realistic ATP production, while also significantly slowing down the circuit simulation due to excessive coupling steps. Moreover, the choice of coupling time needs to account for the heterogeneous response of neurons, as different me-types lead to neurons consuming and producing ATP at varying rates depending on their electrophysiological behavior.

Therefore, the coupling time was chosen to balance these factors: it needed to be long enough to minimize computational costs while maintaining solver stability and neurophysiological plausibility, ensuring that ATP concentrations remain above zero. After investigating ATP

production and consumption dynamics through simulations (see **Reviewer Response Figure 4** and **Figure 5**), we determined that a 100 ms coupling time offers the optimal trade-off. The choice is confirmed by the average ATP production and consumption which are comparable in Figure 2D in the manuscript.

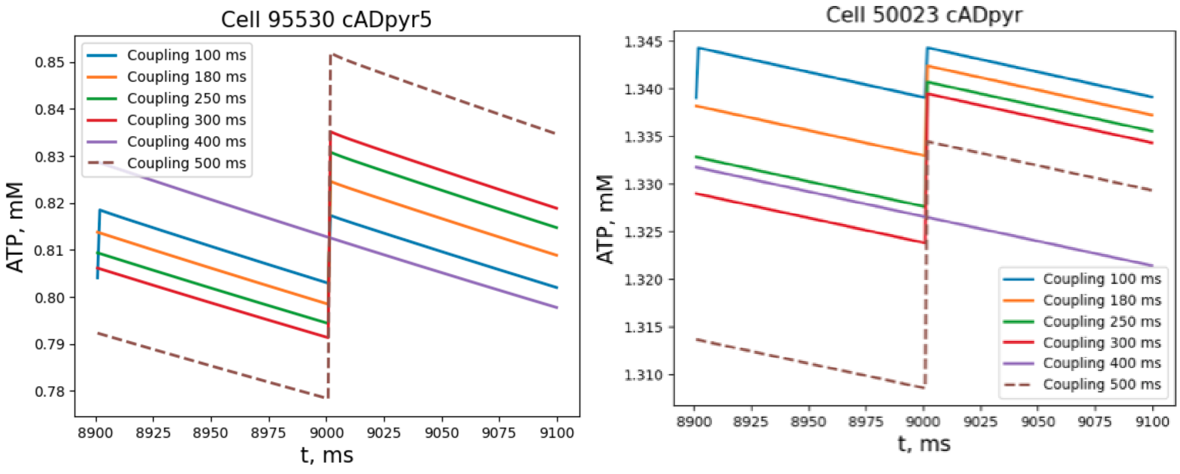

Reviewer Response Figure 4: In a past study, we tested several coupling times ranging from 100 ms to 500 ms in a subcircuit of the microcircuit comprising 10k neurons with final time 10 s. The left panel shows a neuron where, at 9 s, ATP levels appear unaffected by the coupling time. This suggests that the neuron has reached a steady state in ATP production and consumption, making the choice of coupling time less critical. In contrast, the right panel depicts a cell where a 100 ms coupling interval helps maintain a higher ATP level, better balancing consumption. Conversely, coupling every 500 ms leads to greater variability in ATP consumption and production, likely because the neuron depletes ATP too quickly. Ideally, the model should achieve a stable balance between ATP consumption and production, which is why we chose a 100 ms coupling time. Note: this study was done before we re-optimised the e-models.

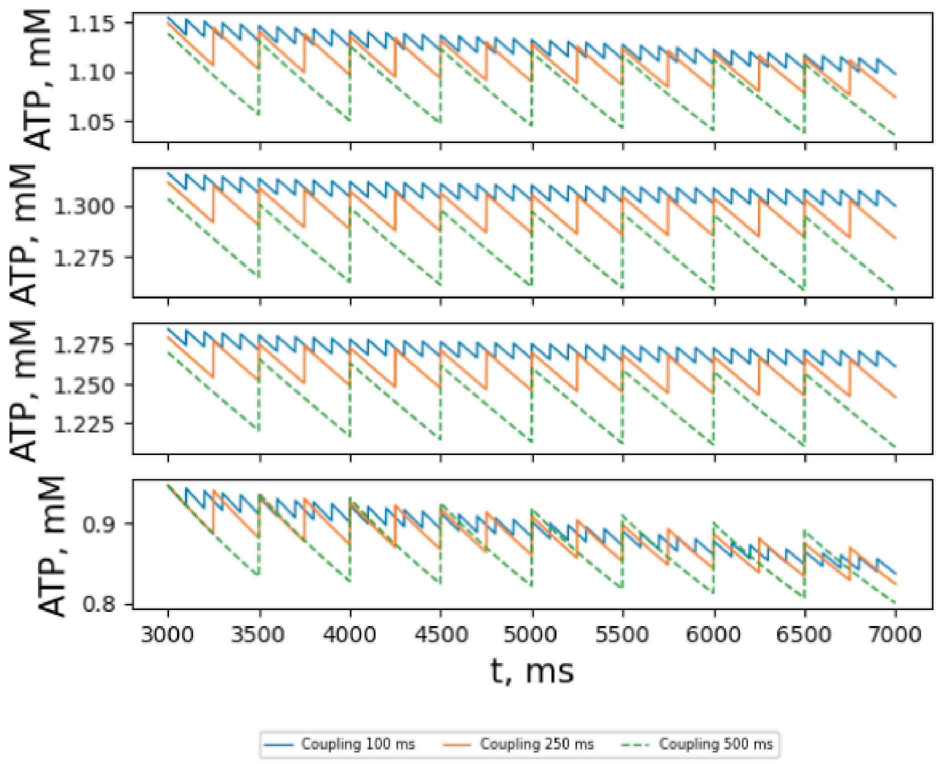

Reviewer Response Figure 5: This figure builds on the microcircuit study presented in Reviewer Response Figure 4, observing the time period from 3 s to 7 s. We believe this plot further supports our choice of a 100 ms coupling time, as it results in smoother ATP dynamics with less fluctuation and a better balance between production and consumption.

Manuscript changes were made to clarify the choice of 100 ms, specifically in L. 99-102.

4. For clarity and fairness, I think you should mention somewhere in the beginning of the article that you only model metabolic ageing and you do not include any morphological changes attributed to ageing. This was not obvious to me until I reached the discussion, and I think it is essential for general readers.

To improve clarity, we have added a sentence in the Introduction to clearly state that our focus is solely on metabolic ageing (L. 65). Additionally, we have included a sentence in the Results section (L. 300-301) to further clarify it.

5. It would also be very nice if in the discussion you could provide a hypothesis of how you think changes due to ageing in the morphology could affect your results.

Thank you for your suggestion, we have added insights in the discussion on how neuronal morphological changes due to ageing could affect our results (L. 430-434). We acknowledge that this discussion could be further expanded to include astrocytic morphological changes,

shifts in the ratio of inhibitory and excitatory neurons, and changes in astrocyte numbers—important aspects for future work.

6. On page 4, you mention that the volume fraction of the astrocyte is included in the system of differential equations. Changes in volume fractions during different pathologies such as ischemia and cortical spreading depression have been very well documented in both experimental and computational literature. I was wondering whether you also consider the changes in the extracellular space. Additionally, do these volume fractions also change for the ageing model proposed?

We agree that the volume fraction of astrocytes is likely to change with ageing, and we also recognize that ageing impacts other factors such as neuronal population numbers and the balance between excitatory and inhibitory neurons. However, in this model, we have focused specifically on metabolic changes and have not incorporated structural alterations in circuit composition, such as potential reductions in astrocyte or neuron numbers, which would affect their respective volume fractions. This is certainly an interesting area for further investigation, and we plan to consider it in future work.

Regarding the extracellular space, our model includes a volume fraction parameter that accounts for glucose transport between compartments, including the capillary, neurons, and astrocytes, passing through the interstitium, basal lamina, and endothelium. These parameters, however, do not change with ageing in the current model. Future developments could further refine the model of the extracellular space to provide a more realistic description of ion diffusion dynamics, including potassium, calcium, and others.

7. I believe not dynamically modelling blood flow is the most significant limitation of your model and might strongly affect your results. Including a blood flow model would increase complexity even further and would add one more timescale to account for. This has actually been done before in https://www.sciencedirect.com/science/article/abs/pii/S0022519319302309 where the authors coupled an electro-metabolic model with a hemodynamic one in which the blood flow is tracked through arteries, capillaries and veins. There is also large literature on hemodynamic models and a lot of experimental data available on rats that could potentially aid you in developing your exciting future work.

We thank the reviewer for pointing out this interesting article. We have added a sentence in the discussion to highlight this point, which will be very useful for our future work (L. 421-423).

We would like to sincerely thank the reviewers once again for their thorough evaluation and valuable suggestions, which have significantly contributed to improving the manuscript. We hope the reviewers are satisfied with the revisions and find the updated manuscript to be significantly improved.

Kind regards,
The authors
